# Highly conserved extracellular residues mediate interactions between pore-forming and regulatory subunits of the yeast Ca^2+^ channel related to the animal VGCC/NALCN family

**DOI:** 10.1074/jbc.RA120.014378

**Published:** 2020-07-20

**Authors:** Takuto Hayashi, Keita Oishi, Midori Kimura, Kazuko Iida, Hidetoshi Iida

**Affiliations:** 1 Department of Biology, Tokyo Gakugei University, 4-1-1 Nukuikita-machi, Koganei, Tokyo, Japan; 2 Laboratory of Biomembrane, Tokyo Metropolitan Institute of Medical Science, 2-1-6 Kamikitazawa, Setagaya, Tokyo, Japan

**Keywords:** voltage-gated calcium channel, VGCC, sodium leak channel nonselective, NALCN, calcium influx, subunit interaction, protein-protein interaction, yeast, Saccharomyces cerevisiae, calcium channel, structure-function

## Abstract

Yeasts and fungi generate Ca^2+^ signals in response to environmental stresses through Ca^2+^ channels essentially composed of Cch1 and Mid1. Cch1 is homologous to the pore-forming α_1_ subunit of animal voltage-gated Ca^2+^ channels (VGCCs) and sodium leak channels nonselective (NALCNs), whereas Mid1 is a membrane-associated protein similar to the regulatory α_2_/δ subunit of VGCCs and the regulatory subunit of NALCNs. Although the physiological roles of Cch1/Mid1 channels are known, their molecular regulation remains elusive, including subunit interactions regulating channel functionality. Herein, we identify amino acid residues involved in interactions between the pore-forming Cch1 subunit and the essential regulatory Mid1 subunit of *Saccharomyces cerevisiae*. *In vitro* mutagenesis followed by functional assays and co-immunoprecipitation experiments reveal that three residues present in a specific extracellular loop in the repeat III region of Cch1 are required for interaction with Mid1, and that one essential Mid1 residue is required for interaction with Cch1. Importantly, these residues are necessary for Ca^2+^ channel activity and are highly conserved in fungal and animal counterparts. We discuss that this unique subunit interaction-based regulatory mechanism for Cch1 differs from that of VGCCs/NALCNs.

Animal voltage-gated Ca^2+^ channels (VGCCs) are a family of multimeric transmembrane proteins composed of a pore-forming α_1_ subunit and three auxiliary subunits (α_2_/δ, β, and γ), and interactions between the α_1_ subunit and the three subunits are important for trafficking to the plasma membrane and functional modulation (1). Although animal VGCCs in excitable cells function as essential regulators of nerve activity and muscle contraction, the functions of their counterparts in yeast cells remains largely under-investigated, although they are known to play essential roles in responses to environmental stresses instead of voltage changes.

The yeast Ca^2+^ channel is composed mainly of two essential subunits; Ca^2+^ channel homolog 1 (Cch1) and mating pheromone-induced death 1 (Mid1). Cch1 is a homolog of the α_1_ subunit of animal VGCCs and recently characterized Na^+^ leak channels nonselective (NALCNs) (2–4), but the amino acid sequence identity between Cch1 and animal VGCCs and NALCNs is low. For example, Cch1 of the yeast *Saccharomyces cerevisiae* shares only 24% amino acid sequence identity with mammalian L-type VGCCs, even though Cch1 shares several structural features of the α_1_ subunit, including molecular size, putative transmembrane topology, and domain structure (2). Specifically, like α_1_ subunits, the Cch1 subunit is composed of four repeats (I, II, III, and IV), each of which has six transmembrane segments (S1−S6) and a pore-forming region located between S5 and S6. However, unlike α_1_ subunits, the S4 segment of the repeat IV region in Cch1 lacks a set of voltage-sensing, positively-charged arginine or lysine residues, whereas Cch1 repeats I−III possess four sets of positively-charged amino acid residues (2). This deficiency in arginine and lysine residues could explain the lack of voltage dependence of Ca^2+^ currents observed for *Cryptococcus neoformans* Cch1 (5, 6) and is consistent with findings that the *S. cerevisiae* Ca^2+^ channel mainly composed of Cch1 and Mid1 is activated to permeate Ca^2+^ by environmental stimuli, including mating pheromones (2, 3, 7), endoplasmic reticulum (ER) stress (8), alkaline stress (9), cold stress (10), hyperosmotic stress (11), ethanol stress (12), and mechanical stress (13, 14).

Mid1 is an α_2_/δ-like protein (7, 15). Although nonhomologous to the α_2_/δ subunit of animal VGCCs at the amino acid sequence level, Mid1 possesses several structural features of animal α_2_/δ subunits, including an N-terminal signal peptide, a number of *N*-glycosylation sites, a Cys-rich domain, and extracellular localization (7, 15, 16). The Cys-rich region is of interest because it is highly conserved in Mid1 superfamily proteins in yeasts, fungi, and animals (17).

Although animal α_2_/δ subunits are not required for Ca^2+^-permeability of α_1_ subunits but are required for membrane targeting and gating properties of α_1_ subunits (18), Mid1 is indispensable for the ability of Cch1 to permeate Ca^2+^; in the absence of Mid1, Cch1 is completely unable to mediate Ca^2+^ influx (2, 3, 7). Therefore, interaction between Cch1 and Mid1 is essential for Ca^2+^ channel activity. Likewise, the NLF-1 and dmeMid1(CG33988) subunits of animal NALCNs, which contain Cys-rich regions homologous to those of Mid1, are absolutely required for the activity of NALCNs (17, 19). Fungi possess the γ subunit homolog Ecm7 that has a weak positive effect on the activity of the Cch1/Mid1 Ca^2+^ channel in WT *S. cerevisiae* cells (15, 20). However, genes encoding β subunit homologs have not been found in fungal genomes. Thus, it is crucial to investigate the interaction between Cch1 and Mid1 to better understand the structural and functional properties of fungal Ca^2+^ channels.

The interaction between mammalian α_1_ subunits and α_2_/δ subunits has been successfully explored using electrophysiological and cell biological approaches. α_2_/δ Subunits play a role in increasing Ca^2+^ current density, mainly by enhancing the trafficking of α_1_ subunits to augment the density of VGCC complexes in the plasma membrane (21, 22). In addition, recent single-particle cryo-EM experiments provided detailed information on the rabbit VGCC (Ca_v_1.1) complex, revealing multiple interaction sites between the extracellular loops of repeats I−III of the α_1_ subunit and the α_2_/δ subunit (23, 24). In particular, the extracellular loops connecting the S5 segment and the first half of the pore loop in repeats II and III (designated L5_II_ and L5_III_, respectively) and the extracellular loop between the S1 and S2 segments of repeat I (designated L1-2_I_) were found to interact with a specific region of the α_2_/δ subunit (24). By contrast, interactions between Cch1 and Mid1 have not yet been characterized, even though they are essential for the Ca^2+^ permeation ability of Cch1.

In the present study, we identified extracellular amino acid residues required for the interaction between Cch1 and Mid1 of *S. cerevisiae*. We focused on extracellular Cys residues of Cch1 and Mid1 because those of Cch1 are conserved in the Cch1/VGCC/NALCN family, and because Mid1 also has a conserved Cys-rich region. Alanine scanning mutagenesis of eight conserved, extracellular Cys residues of Cch1 revealed that two in repeat III are required for interaction with Mid1. Furthermore, PCR-based random mutagenesis of Cch1 repeat III identified three additional amino acid residues necessary for interaction with Mid1. Furthermore, one of 12 Cys residues in the Cys-rich region of Mid1 is required for interaction with Cch1. Based on these findings, we discuss the diversity and universality of the interaction between Cch1 and Mid1 subunits in yeast and their mammalian counterparts.

## Results

### Eight extracellular Cys residues are conserved in the pore-forming subunits of Cch1/VGCC/NALCN family members

**Figure 1.**
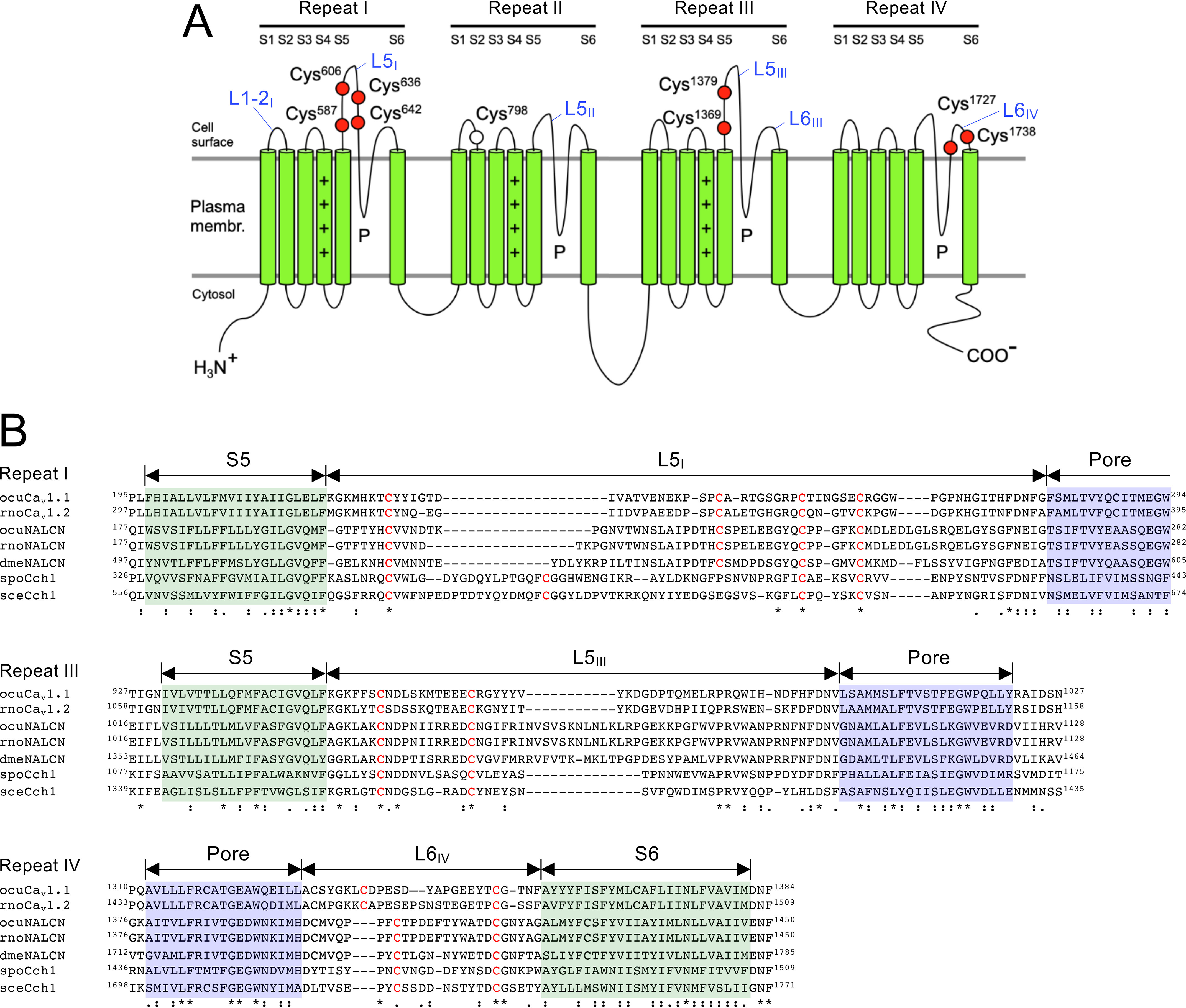
**Structure of the Cch1 protein and its homologous VGCC/NALCN proteins. *A***, position of extracellular Cys residues in the Cch1 protein. The transmembrane topology was determined by manual assertion deduced from a combination of prediction using the transmembrane topology web server TOPCONS (25) and cryo-EM analysis of the rabbit VGCC Ca_v_1.1 (23, 24). For details, see the main text. All nine extracellular Cys residues present in Cch1 are represented by *circles*. *Red circles* indicate Cys residues conserved in the Cch1/VGCC/NALCN family in yeasts, fungi, and animals. *Open circles* indicate nonconserved Cys residues. *Plus signs* in the S4 segments indicate conserved, positively-charged amino acids. ***B***, multiple amino acid sequence alignment of the S5-loop-pore region in repeats I and III and the pore-loop-S6 region of repeat IV of the Cch1/VGCC/NALCN family. Note that repeats I, III, and IV include conserved, extracellular Cys residues. Cys residues are colored *red*. The S5 and S6 segments are highlighted with *green boxes*, and the pore regions are highlighted with *purple boxes*. *Asterisks* indicate amino acid identity, and *colons* and *dots* denote conserved and semiconserved variation, respectively. *ocuCav1.1*, *Oryctolagus cuniculus* VGCC α1S (NP_001095190); *rnoCav1.2*, *Rattus norvegicus* VGCC α1C (NP_036649); *ocuNALCN*, *Oryctolagus cuniculus* NALCN (XP_002713059); *rnoNALCN*, *Rattus norvegicus* NALCN (NP_705894); *dmeNALCN*, *Drosophila melanogaster* NALCN (NP_001096981); *spoCch1*, *Schizosaccharomyces pombe* calcium channel (NP_593894); *sceCch1*, *Saccharomyces cerevisiae* calcium channel (NP_011733).

To investigate the interaction between Cch1 and Mid1, we directed attention to the extracellular loops of Cch1. To determine the transmembrane topology of Cch1, we employed a combination of computational approaches using the web server TOPCONS (25) and data from cryo-EM analysis of rabbit VGCC (23, 24). This combination proved essential because the web servers we utilized, including TOPCONS, were unable to predict S4 segments as transmembrane regions due to the presence of four positively-charged Arg or Lys residues in each segment in repeats I−III. Using this combinatorial approach, we determined the transmembrane topology of Cch1 (Fig. 1*A*, Figs. S1 and S2) and identified nine Cys residues in the extracellular loops, eight of which were conserved in the extracellular loops of α_1_ subunits of animal VGCCs and NALCNs, and fungal Cch1 homologs (Fig. 1*B*); four in repeat I (Cys-587, Cys-606, Cys-636, and Cys-642), two in repeat III (Cys-1369 and Cys-1379), and two in repeat IV (Cys-1727 and Cys-1738). Importantly, Cys residues of animal VGCCs corresponding to the eight conserved Cys residues are involved in intra-loop disulfide bonding (24) and are necessary for ion channel integrity (26). One of the nine Cys residues in repeat II, Cys-798, is not conserved, even in yeasts and fungi, and was not subsequently examined. Note that the transmembrane topology model is completely identical to those of animal VGCCs and NALCNs and partly differs from the previously proposed model of Cch1, in which the four Cys residues in repeat I is located in the cytoplasm (27).

**Figure 2.**
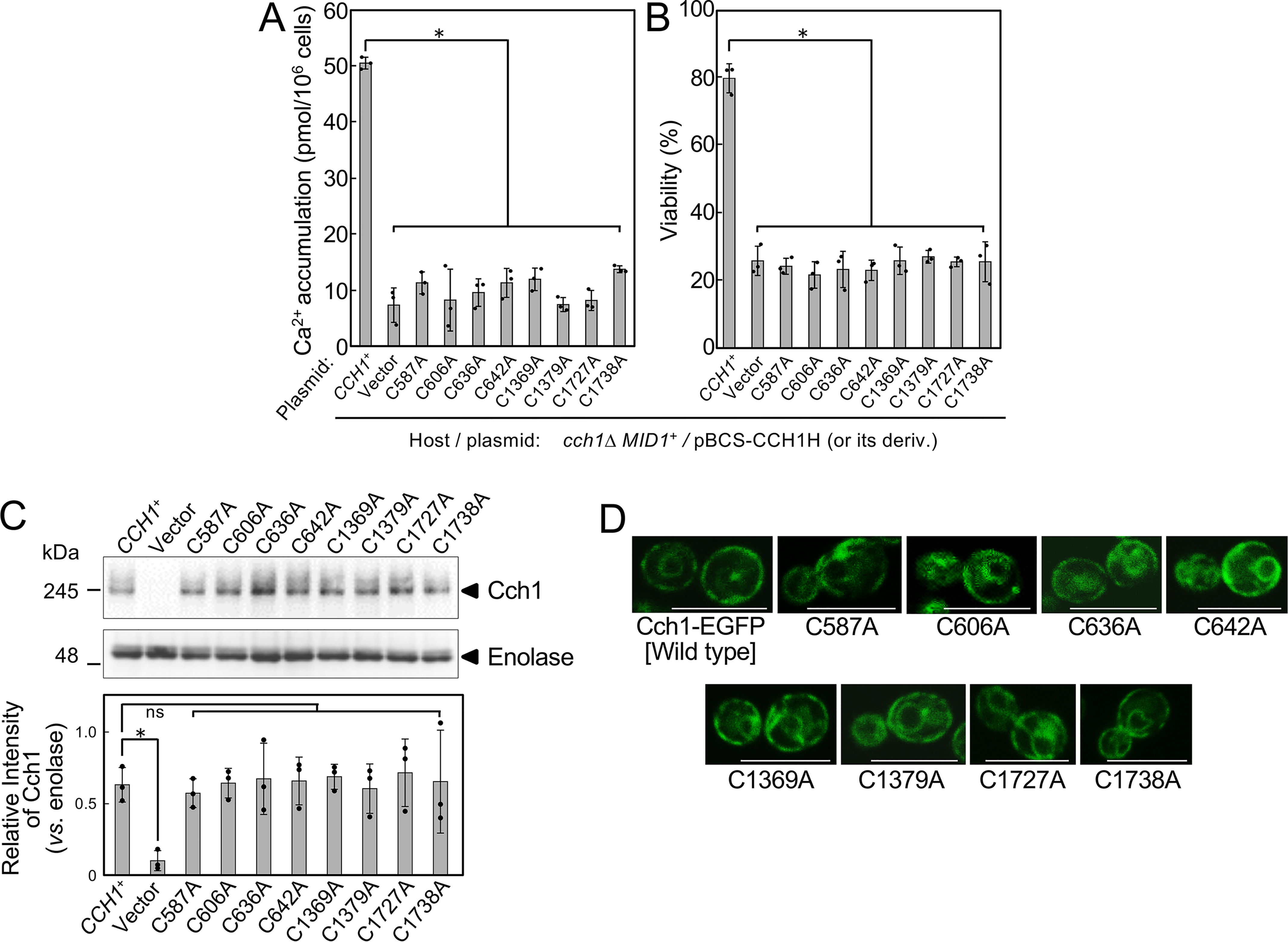
**Functional importance of extracellular Cys residues in the Cch1 protein. *A***, Ca^2+^ accumulation in the *cch1*Δ mutant transformed with a low-copy plasmid expressing a mutant Cch1 protein with a Cys to Ala substitution under the control of the *CCH1* promoter. Substitutions were introduced into the *CCH1* gene in the low-copy plasmid pBCS-CCH1H. Cells containing pBCS-CCH1H or its derivatives were incubated for 2 h with 6 μm α-factor in SD.Ca100 medium and examined for Ca^2+^ accumulation. Mean ± S.D. (*error bars*) from three independent experiments are presented. Data were analyzed by ordinary one-way ANOVA (*F*(9,20) = 76.77, *p* = 55e-13) followed by Dunnett's post hoc test (*, *p* < 1e-10, mutant *versus CCH1*^+^). ***B*,** viability of the *cch1*Δ mutant transformed with the low-copy plasmids described above. Transformants were incubated for 8 h with 6 μm α-factor in SD.Ca100 medium and examined for cell viability. Mean ± S.D. (*error bars*) from three independent experiments are presented. Data were analyzed by ordinary one-way ANOVA (*F*(9,20) = 60.12, *p* = 61e-12) followed by Dunnett's post hoc test (*, *p* < 1e-10, mutant *versus CCH1*^+^). ***C***, Western blotting analysis showing that the cellular abundance of Cys-Ala substitution mutant proteins is comparable with that of WT Cch1. The cells employed were the same as those used above. Whole-cell extracts were subjected to SDS-PAGE followed by Western blotting with polyclonal anti-Cch1 (*upper panel*) and anti-enolase antibodies (*middle panel*). Enolase is an internal loading marker. Typical results from three independent experiments are shown. The *lower panel* shows the relative amount of proteins on three independent Western blots, one of which is presented in the above panels. The intensity of individual bands was quantified with the ImageJ software after capturing Western blotting images in the ChemiDoc XRS+ imaging system (Bio-Rad) and calculated relative to the intensity of enolase used as an internal standard in each sample. Mean ± S.D. from three independent Western blots are presented. Data were analyzed by ordinary one-way ANOVA (*F*(9,20) = 2.697, *p* = 0.031) followed by Dunnett's post hoc test (*, *p* < 0.05, *ns*, not significant, *p* > 0.997, mutant *versus CCH1*^+^). ***D***, confocal fluorescence microscopy image showing that the subcellular localization of Cch1-EGFP and its Cys-Ala substitution mutant proteins are essentially the same. The *cch1*Δ cells harboring pBCT-CCH1H-EGFP or its derivatives were subjected to confocal imaging. Typical results from three independent experiments are shown. *Scale bars* = 10 μm.

### All eight extracellular Cys residues are important for Cch1 activity

To examine the contribution of the eight Cys residues to channel activity, we performed alanine scanning mutagenesis studies. Low-copy plasmids carrying *CCH1* promoter:*CCH1* genes with Cys-to-Ala substitutions were constructed and used to transform the *cch1*Δ mutant defective in both cell viability maintenance and Ca^2+^ accumulation activity after exposure to the mating pheromone α-factor, an activator of the Cch1/Mid1 channel (2, 3, 7). We then assessed the Ca^2+^ permeability of the resulting mutant Cch1 proteins by measuring Ca^2+^ accumulation in the transformants after exposure to α-factor. Fig. 2*A* shows that all eight substitution mutants (C587A, C606A, C636A, C642A, C1369A, C1379A, C1727A, and C1738A) showed low Ca^2+^ accumulation comparable with the *cch1*Δ mutant carrying an empty vector, suggesting that all eight proteins had lost Ca^2+^ permeation activity. Based on the previous observation that Ca^2+^ accumulation activity is quantitatively correlated with the viability of *cch1* mutants (16, 28), we also assessed the viability of the transformants after exposure to α-factor, and as expected, the results supported the same conclusions (Fig. 2*B*).

It is conceivable that the apparent loss of Ca^2+^ permeation activity by the mutant Cch1 proteins could be due to low cellular content and/or mislocalization. To examine this possibility, we evaluated the cellular content for each of the proteins by Western blotting. Fig. 2*C* shows that the cellular content for each of the eight mutant Cch1 proteins was similar to that of WT Cch1. To analyze the subcellular localization of the mutant Cch1 proteins, we fused the gene encoding enhanced GFP (EGFP) to the C terminus in the low-copy plasmid carrying the strong *TDH3* promoter (29), which allowed high-level expression of the fused Cch1-EGFP proteins, thereby enabling us to observe their subcellular localization by fluorescent microscopy (28). Fig. 2*D* shows that all eight mutant Cch1 proteins tagged with EGFP were localized at the plasma membrane and the ER membrane, similar to the WT Cch1-EGFP protein. This subcellular localization is consistent with previous reports (30). Thus, the above possibility is unlikely. The ER localization of the Cch1-EGFP proteins may be due to their overexpression under the control of the *TDH3* promoter (28, 31). Taken together, the results indicate that all eight conserved extracellular Cys residues are required for Cch1 activity.

### Cch1 mutants C1369A and C1379A possess weak Ca^2+^ permeability activity and overexpression does not interfere with WT Cch1

**Figure 3.**
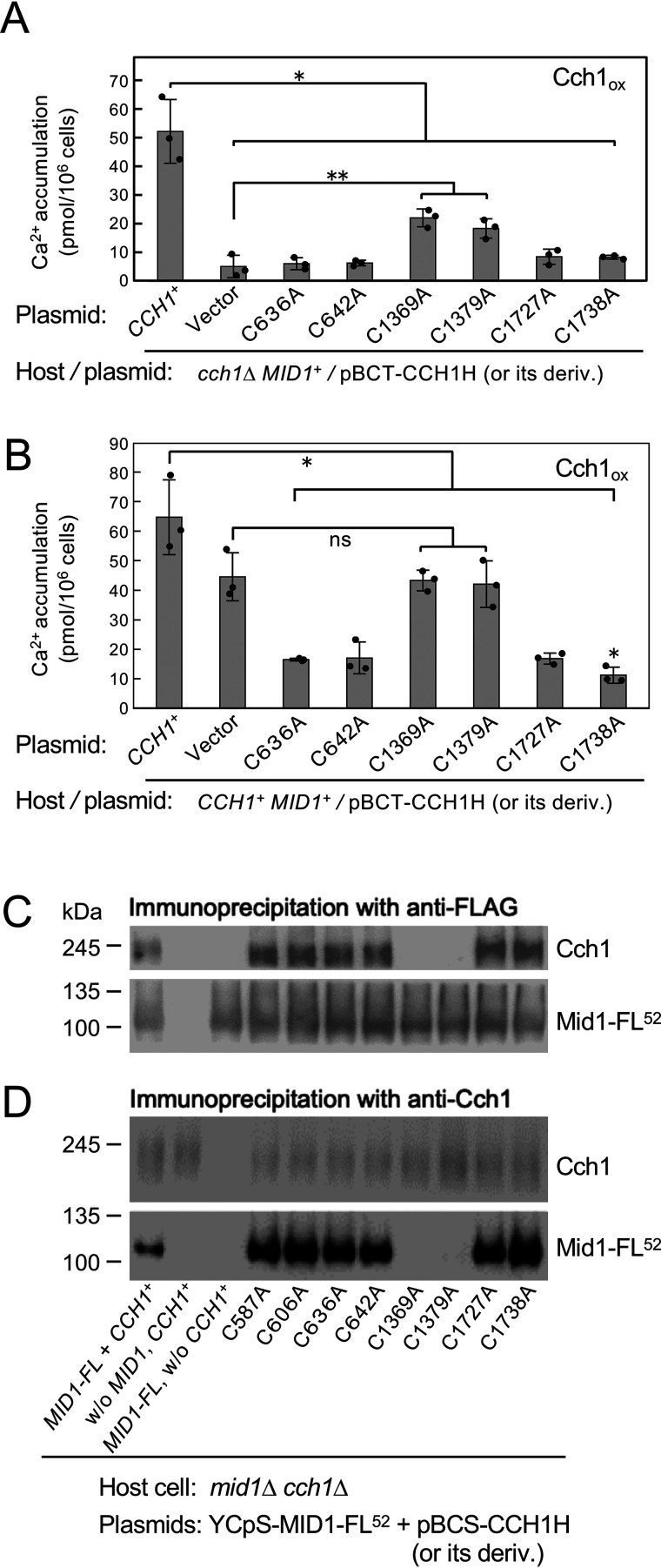
**Two extracellular Cys residues of Cch1 are involved in the interaction with Mid1. *A***, Ca^2+^ accumulation in the *cch1*Δ mutant transformed with a plasmid overexpressing a mutant Cch1 protein (designated Cch1_ox_ in the figure) with a Cys to Ala substitution under the control of the *TDH3* promoter. Substitutions were introduced into the *CCH1* gene in the plasmid pBCT-CCH1H containing the *TDH3* promoter. Ca^2+^ accumulation assays were performed as described in the legend to Fig. 2*A*. Mean ± S.D. (*error bars*) from three independent experiments are presented. Data were analyzed by ordinary one-way ANOVA (*F*(7,16) = 35.47, *p* = 39e-08) followed by Dunnett's post hoc test (*, *p* < 1e-6, mutant *versus CCH1*^+^; **, *p* < 0.02, mutant *versus* vector). ***B***, Ca^2+^ accumulation in WT (*CCH1*^+^) cells transformed with a plasmid overexpressing a mutant Cch1 protein with a Cys to Ala substitution under the control of the *TDH3* promoter. Amino acid substitution and Ca^2+^ accumulation assays were the same as above. Mean ± S.D. (*error bars*) from three independent experiments are presented. Data were analyzed by ordinary one-way ANOVA (*F*(7,16) = 25.93, *p* = 37e-07) followed by Dunnett's post hoc test (*, *p* < 0.006, mutant *versus CCH1*^+^; *ns,* not significant, *p* > 0.99, mutant *versus* vector). ***C***, Cch1-C1369A and -C1379A proteins do not co-immunoprecipitate with Mid1-FL^52^. Half of the whole-cell extracts prepared from the *mid1*Δ *cch1*Δ mutant expressing Mid1-FL^52^ together with Cch1 or its derivatives was immunoprecipitated with anti-FLAG antibody that binds to Mid1-FL^52^. Precipitates were subjected to SDS-PAGE followed by Western blotting with either anti-FLAG antibody or anti-Cch1 antibodies. Typical results from three independent experiments are shown. ***D***, Mid1-FL^52^ does not co-immunoprecipitate with Cch1-C1369A and -C1379A proteins. The other half of the whole-cell extracts were treated as described above, but instead subjected to immunoprecipitation with anti-Cch1 antibodies. Typical results from three independent experiments are shown.

The above results do not necessarily indicate that all eight Cys-Ala mutations are loss-of-permeability mutations; it is also possible that some of the mutations reduce the ability of the mutant Cch1 proteins to interact with Mid1 while maintaining Ca^2+^ permeability. To examine this possibility, we employed a protein overexpression approach. Because the Cch1–Mid1 interaction must be dependent on the stoichiometry of both proteins, overexpression of mutant Cch1 proteins with reduced interacting ability might improve the Ca^2+^ accumulation activity of the *cch1*Δ mutant, which possesses one copy of the genomic *MID1*^+^ gene. This experiment could identify extracellular Cys residues that take part in the interaction with Mid1. We therefore constructed plasmids from which the eight *cch1* mutant genes described above were transcribed under the control of the *TDH3* promoter, and transformed the *cch1*Δ mutant separately with each plasmid. Under our experimental conditions, the *TDH3* promoter resulted in a ∼50-fold increase in Cch1 protein abundance compared with the *CCH1* promoter (28). We first examined the viability of the eight transformants and found that C1369A and C1379A proteins exhibited a weak but significant ability to enhance the viability of the *cch1*Δ mutant, whereas the other six (C587A, C606A, C636A, C642A, C1727A, and C1738A) showed no activity (data not shown). Essentially the same results were obtained for Ca^2+^ accumulation experiments; as shown in Fig. 3*A*, the C1369A and C1379A proteins displayed higher Ca^2+^ permeation activity than the control (vector), whereas C636A, C642A, C1727A, and C1738A proteins showed no activity. Based on the results of both viability and Ca^2+^ accumulation experiments, we suggest that C587A, C606A, C636A, C642A, C1727A, and C1738A mutations are loss-of-permeability mutations.

The above results also suggest that C1369A and C1379A proteins possess Ca^2+^ permeability activity but lack the full ability to interact with Mid1, whereas C587A, C606A, C636A, C642, C1727A, and C1738A proteins do not possess Ca^2+^ permeability activity but may be able to interact with Mid1, and this interaction would render these six proteins able to compete with WT Cch1 to interact with Mid1. Therefore, it is possible that overexpression of the former two proteins in *CCH1*^+^ cells may not interfere with the ability of WT Cch1 to interact with Mid1, whereas overexpression of the other six proteins may interfere. To examine this possibility, we overexpressed six of the eight mutant Cch1 proteins in WT (*CCH1*^+^ *MID1*^+^) cells. Fig. 3*B* shows that C1369A and C1379A proteins did not interfere with the function of WT Cch1, whereas C636A, C642A, C1727A, and C1738A proteins did interfere. We speculate that the former two mutant proteins cannot compete with WT Cch1 to interact with Mid1, and thus do not interfere with the function of WT Cch1. Taken together, the results suggest that Cys-1369 and Cys-1379 of Cch1 are involved in the interaction with Mid1.

### Cch1 mutants C1369A and C1379A do not co-immunoprecipitate with Mid1

To confirm the above hypothesis, we conducted co-immunoprecipitation experiments using *cch1*Δ *mid1*Δ cells co-expressing Mid1 tagged internally with FLAG (Mid1-FL^52^) and each of the eight mutant Cch1 proteins, both of which were expressed from their own promoters. Whole-cell extracts of co-expressing cells were immunoprecipitated by anti-FLAG antibody and precipitates were analyzed by SDS-PAGE followed by Western blotting with anti-Cch1 antibody and anti-FLAG antibody.

Fig. 3*C* shows that C1369A and C1379A mutant Cch1 proteins did not co-precipitate with Mid1-FL^52^, whereas the other six mutant Cch1 proteins (C587A, C606A, C636A, C642A, C1727A, and C1738A) did co-precipitate with Mid1-FL^52^, as did WT Cch1. When whole-cell extracts were immunoprecipitated by anti-Cch1 antibody, Mid-FL^52^ did not co-precipitate with Cch1-C1369A or -C1379A (Fig. 3*D*). The above results provide biochemical and physical evidence supporting the conclusion that Cys-1369 and Cys-1379 are required for interaction with Mid1.

### Additional residues in Cch1 repeat III are required for interaction with Mid1

Cys-1369 and Cys-1379 are located in the extracellular loop between segment 5 and the pore in repeat III (L5_III_), suggesting that this loop participates in interaction with Mid1. It is also possible that the neighboring loop between the pore and segment 6 in the same repeat (L6_III_) is involved in the interaction. Therefore, to identify putative Mid1-interacting residues in L5_III_ and L6_III_ regions other than Cys-1369 and Cys-1379, we screened mutated Cch1 proteins that were unable to complement the phenotype of the *cch1*Δ mutant. To do this, we performed error-prone PCR-based random mutagenesis using manganese (32) on a DNA fragment encoding the IIIS3−S6 region. The resulting PCR products were incorporated into an acceptor plasmid using *in vivo* homologous recombination in yeast *cch1*Δ cells (30). The *cch1*Δ cells carrying the reconstructed full-length *cch1* genes on the plasmid were then directly examined for cell viability and Ca^2+^ accumulation activity.

**Figure 4.**
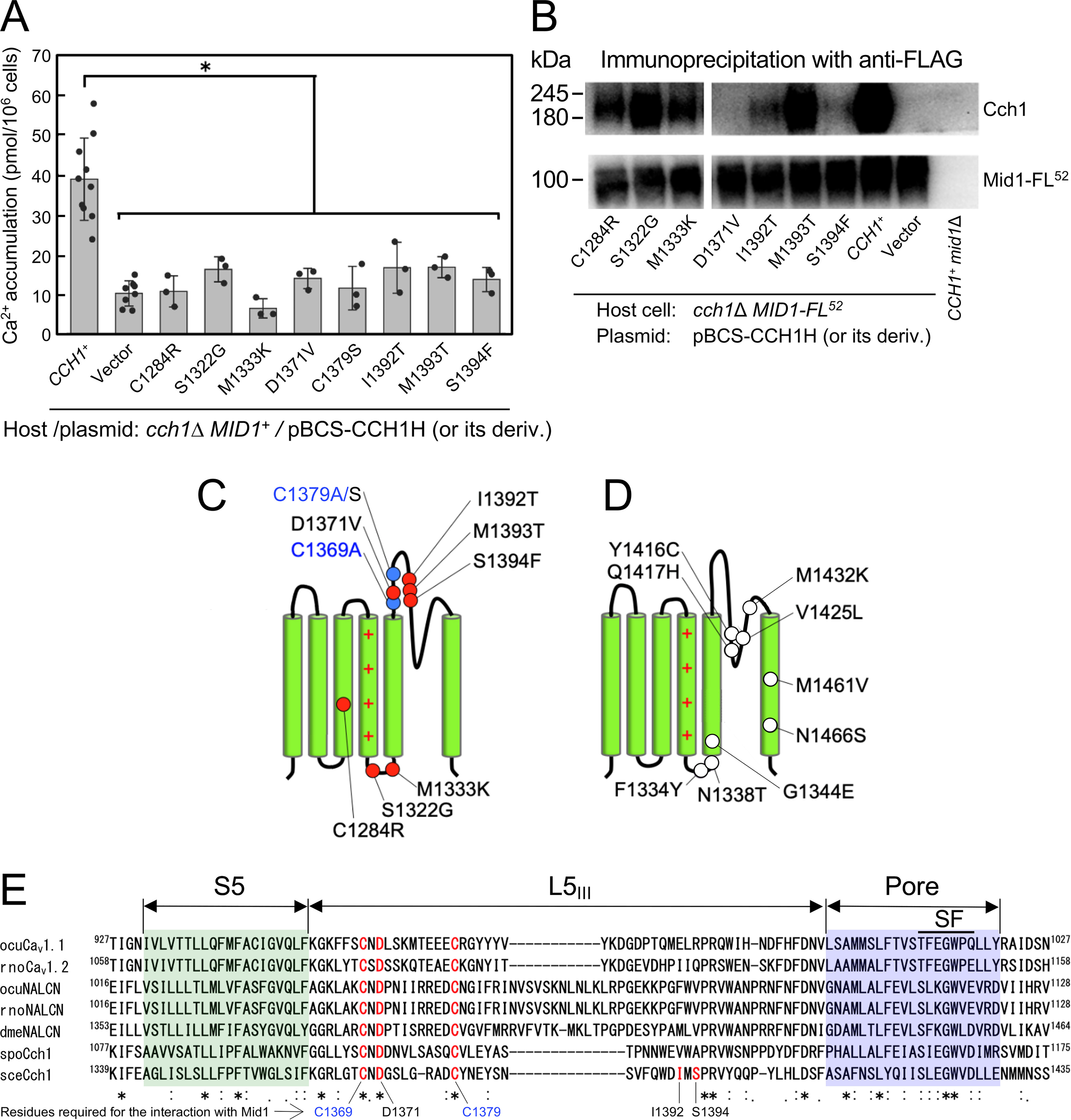
**Random mutagenesis of the IIIS3−S6 region reveals three additional residues involved in the interaction with Mid1. *A***, Ca^2+^ accumulation in the *cch1*Δ mutant transformed with a plasmid carrying a missense mutation in the *CCH1* gene. The mutation was introduced by *in vitro* random mutagenesis and separated from other mutations simultaneously generated by this method. Mutant genes were expressed from the *CCH1* promoter. Ca^2+^ accumulation assays were performed as described in the legend to Fig. 2*A*. Mean ± S.D. (*error bars*) from three independent experiments are presented. Data were analyzed by ordinary one-way ANOVA (*F*(9,32) = 15.67, *p* = 14e-09) followed by Dunnett's post hoc test (*, *p* < 1e-4, mutant *versus CCH1*^+^). ***B***, Cch1 mutants D1371V, I1392T, and S1394F do not co-immunoprecipitate with Mid1-FL^52^. Whole-cell extracts prepared from the *cch1*Δ *MID1-FL^52^* strain expressing one of nine single missense mutations examined above were immunoprecipitated with anti-FLAG antibody, as described in the legend to Fig. 3*C*. Precipitates were subjected to SDS-PAGE followed by Western blotting with either anti-FLAG or anti-Cch1 antibodies. Typical results from three independent experiments are shown. ***C***, schematic representation of the positions of missense mutations in repeat III that cause the loss of Cch1 function. *Red* and *blue circles* represent residues mutated by *in vitro* random mutagenesis and those mutated by Cys-Ala substitution, respectively. C1379A/S are two mutations generated at the same Cys residue. ***D***, schematic representation of the positions of neutral mutations in repeat III caused by the same *in vitro* random mutagenesis as that described above. *Open circles* represent the positions of neutral mutations. ***E***, multiple sequence alignment of the IIIS5−P region. Completely conserved Cys and Asp residues involved in the interaction with Mid1 are shown in *red*. *S. cerevisiae* Cch1-specific Ile and Ser residues involved in the interaction with Mid1 are also colored *red*. The S5 and pore regions are *boxed in green* and *purple*, respectively. The selective filter (SF) region (48, 49) and its putative counterparts are indicated by an *upper bar*. For *asterisks*, *colons*, *dots*, and *abbreviations*, see the legend to Fig. 1*B*.

This strategy enabled us to isolate eight transformants with decreased viability and low Ca^2+^ accumulation (data not shown). Subsequent DNA sequencing of the plasmids isolated from the eight transformants identified eight multiple mutations comprising four double mutations (M1393K/Q1417R; C1379S/Y1416C; M1333K/N1466S; C1284R/M1461V), three triple mutations (C1284R/M1393T/V1425L; F1334Y/I1392T/Q1417H; S1322G/ D1371V/Q1417H), and one quadruple mutation (N1338T/G1344E/S1394F/M1432K). To identify which substitution mutations are responsible for the mutant phenotypes in the multiple mutations, we constructed plasmids in which single mutations were separated from multiple mutations. Phenotypic analysis of *cch1*Δ mutants carrying each of the plasmids identified eight missense mutations (C1284R, S1322G, M1333K, D1371V, C1379S, I1392T, M1393T, and S1394F; Fig. 4*A*). Mutations other than these eight missense mutations were neutral mutations, including F1334Y, N1338T, G1344E, Y1416C, Q1417H, V1425L, M1432K, M1461V, and N1466S (data not shown). Because the missense mutation C1379A, which occupies an identical position to C1379S, was described above, we newly identified seven amino acid residues important for the function of Cch1.

To narrow down possible amino acid residues responsible for the interaction with Mid1, we conducted co-immunoprecipitation assays of the seven mutant proteins. Whole-cell extracts prepared from cells expressing each of the seven mutant proteins together with Mid1-FL^52^ were subjected to immunoprecipitation using anti-FLAG antibody as described above. As shown in Fig. 4*B*, D1371V did not co-precipitate with Mid1-FL^52^ at all, and I1392T and S1394F hardly co-precipitated with Mid1-FL^52^, compared with four of the Cch1 mutant proteins (C1284R, S1322G, M1333K, and M1393T) and WT Cch1. These results suggest that Asp-1371, Ile-1392, and Ser-1394 take part in the interaction with Mid1. To highlight the significance of the amino acid residues within this loop in the interaction with Mid1, we mapped the positions of the missense and neutral mutations in repeat III (Fig. 4, *C* and *D*). It is noteworthy that all three residues (Asp-1371, Ile-1392, and Ser-1394) are located in the L5_III_ loop, similar to the Cys-1369 and Cys-1379 residues identified above.

It is of interest to point out that the Mid1-interacting residues are conserved throughout the Cch1/VGCC/NALCN family. Multiple amino acid alignment of the L5_III_ region showed that three of the five amino acid residues found to be involved in the interaction with Mid1 (Cys-1369, Asp-1371, and Cys-1379) are completely conserved in the Cch1/VGCC/NALCN family covering yeasts to mammals (Fig. 4*E*), suggesting the possibility that these three residues are essential for subunit interactions throughout this family (see “Discussion”).

### Three Cys residues are essential for Mid1 activity

**Figure 5.**
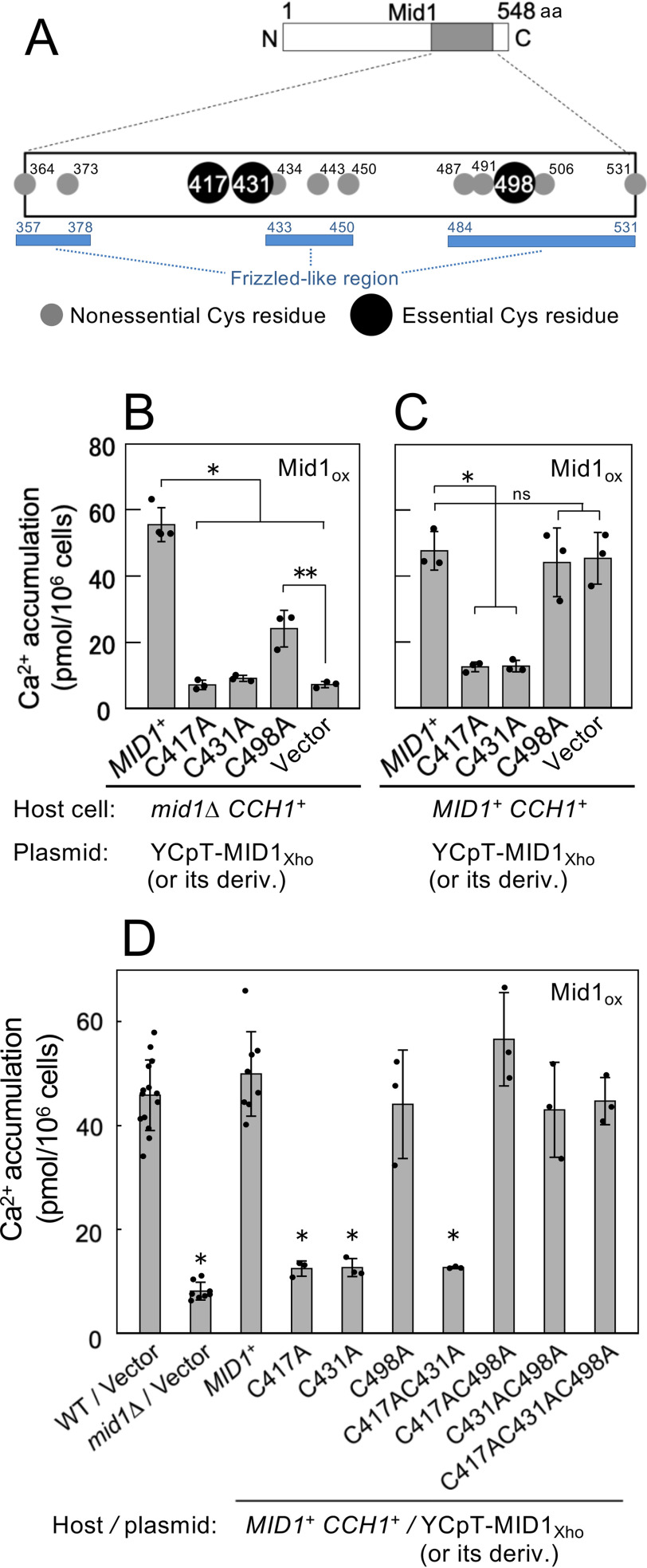
**Position and functional importance of extracellular Cys residues in the Mid1 protein. *A***, schematic representation of the Mid1 protein, highlighting the C-terminal Cys-rich region. *Gray circles* represent Cys residues nonessential for the Ca^2+^-influx–mediating activity of Mid1, and *black circles* denote those essential for activity. *Blue bars* represent the Frizzled-like regions. ***B***, Mid1-C498A possesses Ca^2+^-mediating activity when overexpressed. Ca^2+^ accumulation assays of the *mid1*Δ *CCH1*^+^ mutant transformed with a plasmid overexpressing a mutant Mid1 protein with a Cys to Ala substitution under the control of the *TDH3* promoter were performed as described in the legend to Fig. 2*A*. The substitutions were introduced into the *MID1* gene within the plasmid YCpT-MID1 containing the *TDH3* promoter. Mean ± S.D. (*error bars*) from three independent experiments are presented. Data were analyzed by ordinary one-way ANOVA (*F*(4,11) = 117.7, *p* = 5.98e-09) followed by Dunnett's post hoc test (*, *p* < 1e-6, mutant *versus MID1*^+^; **, *p* < 0.001, mutant *versus* vector). ***C***, Mid1-C498A does not interfere with the function of WT Mid1 when overexpressed. Ca^2+^ accumulation assays of the WT (*MID1*^+^ *CCH1*^+^) strain transformed with the plasmids used in the above experiments were performed as above. Mean ± S.D. (*error bars*) from three independent experiments are presented. Data were analyzed by ordinary one-way ANOVA (*F*(4,10) = 23.72, *p* = 35e-05) followed by Dunnett's post hoc test (*, *p* < 0.001, mutant *versus MID1*^+^; *ns,* not significant, *p* > 0.90, mutant or vector *versus MID1*^+^). ***D***, the C498A mutation cancels the interfering effect of the C417A and C431A mutations on the function of WT Mid1. Ca^2+^ accumulation assays of the WT (*MID1*^+^ *CCH1*^+^) strain transformed with the plasmids used in the above experiments, or those with double or triple mutations, were performed as above. Data for *MID1*^+^, C417A, C431A, C4898A, and vector were adapted from Fig. 5*C* for comparison with the double and triple mutant proteins shown in this figure. Mean ± S.D. (*error bars*) from at least three independent experiments are presented. Data were analyzed by ordinary two-way ANOVA (*F*(9,41) = 43.19, *p* = 2e-16) followed by Dunnett's post hoc test (*, *p* < 1e-4, mutant *versus MID1*^+^).

Mid1 is a plasma and ER membrane protein with 16 *N*-glycosylation sites and a C-terminal Cys-rich region composed of 12 Cys residues (7, 16) (Fig. 5*A*). To identify amino acid residues of Mid1 that interact with Cch1, we focused on the Cys-rich region because it is highly conserved in fungal and animal Mid1 proteins (17), suggesting its functional significance. Previous alanine scanning mutagenesis studies addressed the significance of the 12 Cys residues and revealed that four Cys-Ala substitution mutants (C417A, C431A, C434A, and C498A) did not complement the *mid1* mutation (33). However, analysis of the 12 Cys residues in the present work revealed that one of the four mutant proteins (C434A) was able to complement the *mid1* mutation (data not shown). We found that a one-base deletion leading to a frameshift mutation was present in the previous *MID1-C434A* construct, hence we concentrated on the C417A, C431A, and C498A substitution mutants in subsequent experiments.

### Mid1 C417A and C431A mutants lack Cch1-activating activity, whereas Mid1-C498A lacks Cch1-interacting activity

The inability of the three substitution mutant proteins to complement the *mid1* mutation does not necessarily indicate that these are loss-of-activating function mutations. Alternatively, it is possible that some may retain the ability to activate Cch1 but lack the ability to interact with Cch1. In such cases, overexpression may enhance the ability of such mutant proteins to interact with Cch1. To examine this possibility, we overexpressed each under the control of the *TDH3* promoter in the *mid1*Δ mutant (Fig. 5*B*) and the WT strain (Fig. 5*C*), and measured Ca^2+^ accumulation in the overexpressing cells.

As shown in Fig. 5*B*, *mid1*Δ cells overexpressing C417A or C431A mutants did not exhibit Ca^2+^ accumulation activity, whereas those overexpressing C498A displayed weak activity. Fig. 5*C* shows that WT cells overexpressing C417A or C431A mutants exhibited Ca^2+^ accumulation activity as low as that of the negative control (*mid1*Δ cells bearing an empty vector shown in Fig. 5*B*), whereas WT cells overexpressing the C498A mutant showed Ca^2+^ accumulation activity comparable with that of the positive control (WT cells expressing WT Mid1; Fig. 5*C*). These results suggest that C417A and C431A mutants lack the ability to activate Cch1, but retain the ability to interact with Cch1, and this interacting ability would render these mutant Mid1 proteins able to compete and interfere with the WT Mid1 protein to interact with Cch1. On the other hand, the C498A mutant appeared to retain the ability to activate Cch1, but lose the full ability to interact with Cch1. In other words, Cys-498 may be involved in the interaction with Cch1.

If these suggestions are correct, overexpression of double or triple mutants, such as C417A/C498A, C431A/C498A, and C417A/C431A/C498A would have no effect on the WT Mid1 protein because the C498A mutation would render proteins harboring C417A and C431A unable to compete with and prevent WT Mid1 interacting with Cch1. The results showed that this prediction was correct; although the C417A/C431A and C417A/C431A proteins overexpressed in *MID1*^+^ cells negated the ability of WT Mid1 to mediate Ca^2+^ accumulation, the C417A/C498A, C431A/C498A, and C417A/C431A/C498A proteins did not (Fig. 5*D*). Therefore, we speculate that Cys-498 takes part in the interaction with Cch1.

**Figure 6.**
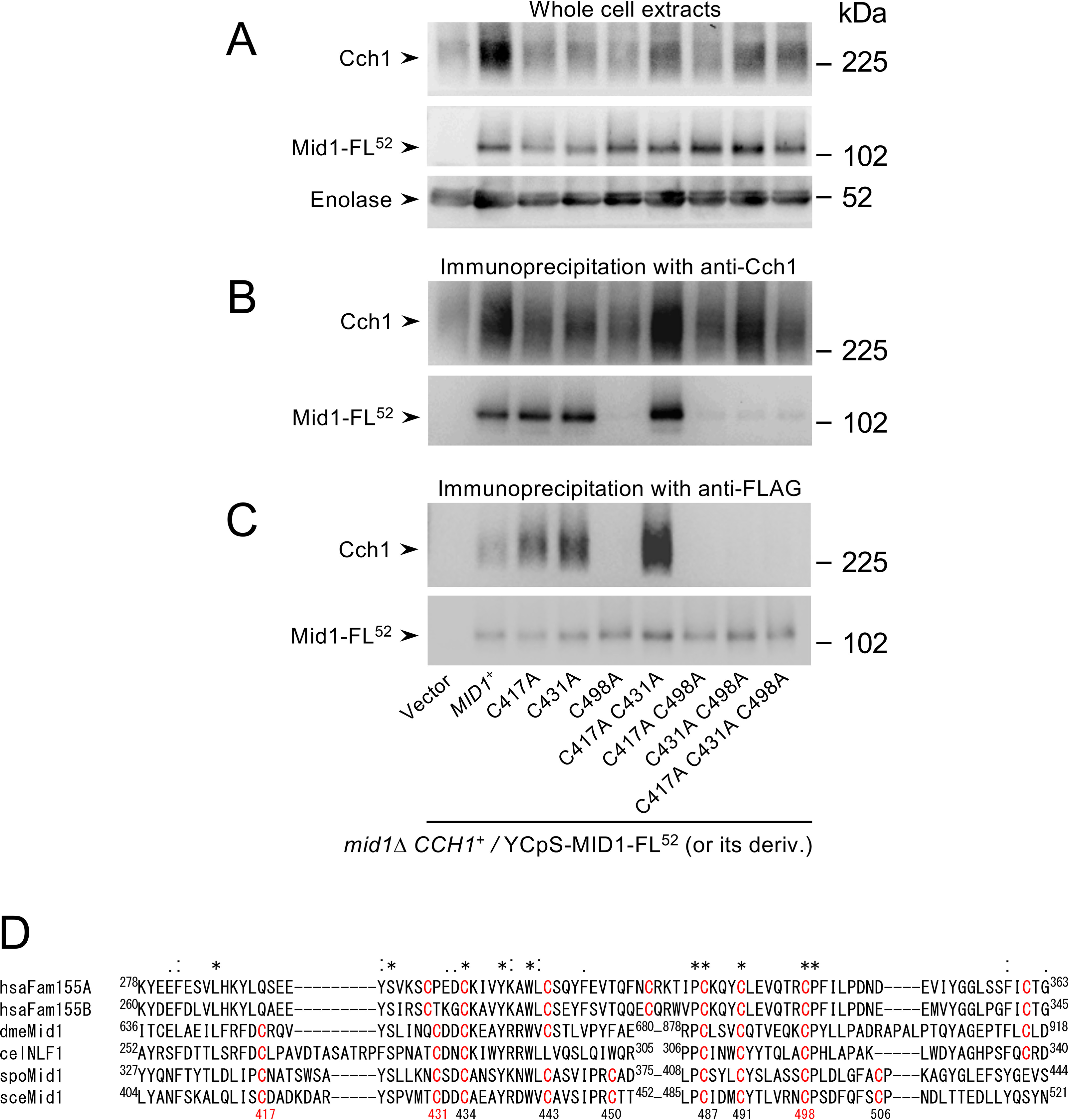
**Mid1-C498A is unable to participate in the interaction with Cch1. *A***, Western blotting of whole-cell extracts. Extracts were prepared from the *mid1*Δ mutant transformed with the plasmid YCpS-MID1-FL^52^ or its derivatives, from which WT Mid1-FL^52^ or its mutants are expressed from the *MID1* promoter, and the extracts was subjected to SDS-PAGE followed by Western blotting with anti-Cch1, anti-FLAG, and anti-enolase antibodies. The blots are indicators of proteins loaded onto the SDS-PAGE gel. ***B***, Mid1-C498A does not co-immunoprecipitate with Cch1. The whole-cell extracts prepared as above were immunoprecipitated with anti-Cch1 and subjected to SDS-PAGE followed by Western blotting with either anti-Cch1 or anti-FLAG antibodies. ***C***, Cch1 does not co-immunoprecipitate with Mid1-C498A. The whole-cell extracts described in *A* were immunoprecipitated with anti-FLAG and subjected to SDS-PAGE followed by Western blotting with either anti-Cch1 or anti-FLAG antibodies. ***D***, multiple amino acid sequence alignment of the Cys-rich domain of *S. cerevisiae* Mid1 and its related proteins. Cys residues are colored *red*. *Numbers under* the alignment refer to the amino acid sequence of sceMid1. The three Cys residues numbered in *red* are essential for the function of Mid1. *Asterisks*, *colons*, and *dots* are described in the legend to Fig. 1*B*. Note that the Cys-rich domain of celNLF presented here is partially inconsistent with that presented in Fig. 1 in the report by Ghezzi *et al.* (17). *hsaFam155A*, *Homo sapiens* Fam155A (NP_001073865); *hsaFam155B*, *H. sapiens* Fam155B (NP_056501); *dmeMid1*, *D. melanogaster* Mid1 (CG33988; NP_001033972); *celNLF1*, *C. elegans* NLF-1 (NP_00135098); *spoMid1*, *Schizosaccharomyces pombe* Mid1 (NP_592865); *sceMid1*, *S. cerevisiae* Mid1 (NP_014108). In ***A*−*C***, typical results from three independent experiments are shown.

To confirm this speculation, we conducted a co-immunoprecipitation assay. Whole-cell extracts were prepared from *mid1*Δ cells carrying the FLAG-tagged mutant and WT Mid1 proteins expressed from the *MID1* promoter (Fig. 6*A*) and immunoprecipitated with anti-Cch1 antibody followed by Western blotting using anti-FLAG antibody. As shown in Fig. 6*B*, neither the C498A protein nor the C417A and C431A proteins carrying the C498A mutation (C417A/C498A, C431A/C498A, and C417A/C431A/C498A proteins) co-precipitated with Cch1, whereas the C417A, C431A, and C417A/C431A proteins co-precipitated like the WT protein. Conversely, when the whole-cell extracts were immunoprecipitated with anti-FLAG antibody followed by Western blotting with the anti-Cch1 antibody, Cch1 did not co-precipitate with C498A, C417A/C498A, C431A/C498A, or C417A/C431A/C498A proteins (Fig. 6*C*). It is noteworthy that Cys-498 is highly conserved, even in animal Mid1-related proteins (Fig. 6*D*).

### C417A, C431A, and C498A mutations do not affect the cellular content or subcellular localization of Mid1

**Figure 7.**
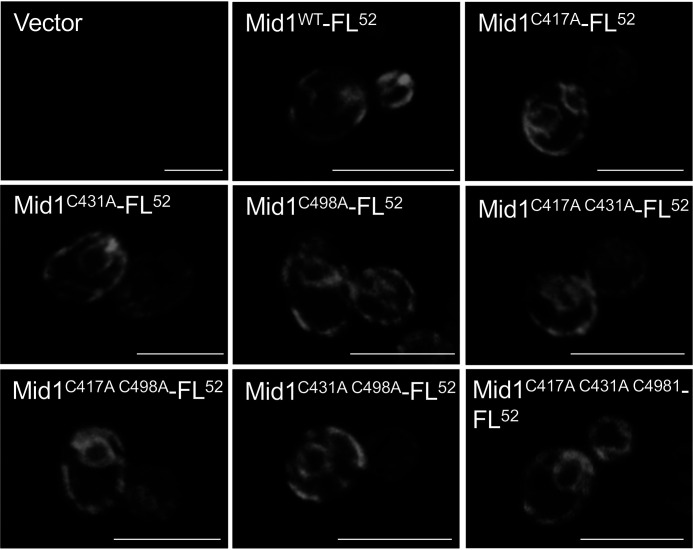
**Subcellular localization of Cys-to-Ala mutant Mid1 proteins.** Cys-to-Ala substitutions do not affect the subcellular localization of Mid1. Cells of the *mid1*Δ mutant transformed with the plasmid YCpS-MID1-FL^52^ or its derivatives as described in the legend to Fig. 6 were subjected to indirect immunofluorescence microscopy. Typical results from three independent experiments are shown. *Scale bars* = 10 μm.

Fig. 6*A* also shows that the abundance of mutant Mid1 proteins was essentially the same as that of the WT protein. In addition, indirect immunofluorescence microscopy indicated that the mutant Mid1 proteins are localized at the plasma and ER membranes, like the WT Mid1 protein (Fig. 7).

Taken together, the results imply that the C498A mutant lacked the ability to interact with Cch1 but retained at least some activity to activate Cch1. By contrast, the C417A and C431A proteins lost the ability to activate Cch1 but retained the ability to interact with Cch1. We therefore conclude that Cys-498 is involved in the interaction with Cch1 and that Cys-417 and Cys-431 participate in the activation of Cch1.

### The Cch1–Mid1 interaction may not involve disulfide bonding

**Figure 8.**
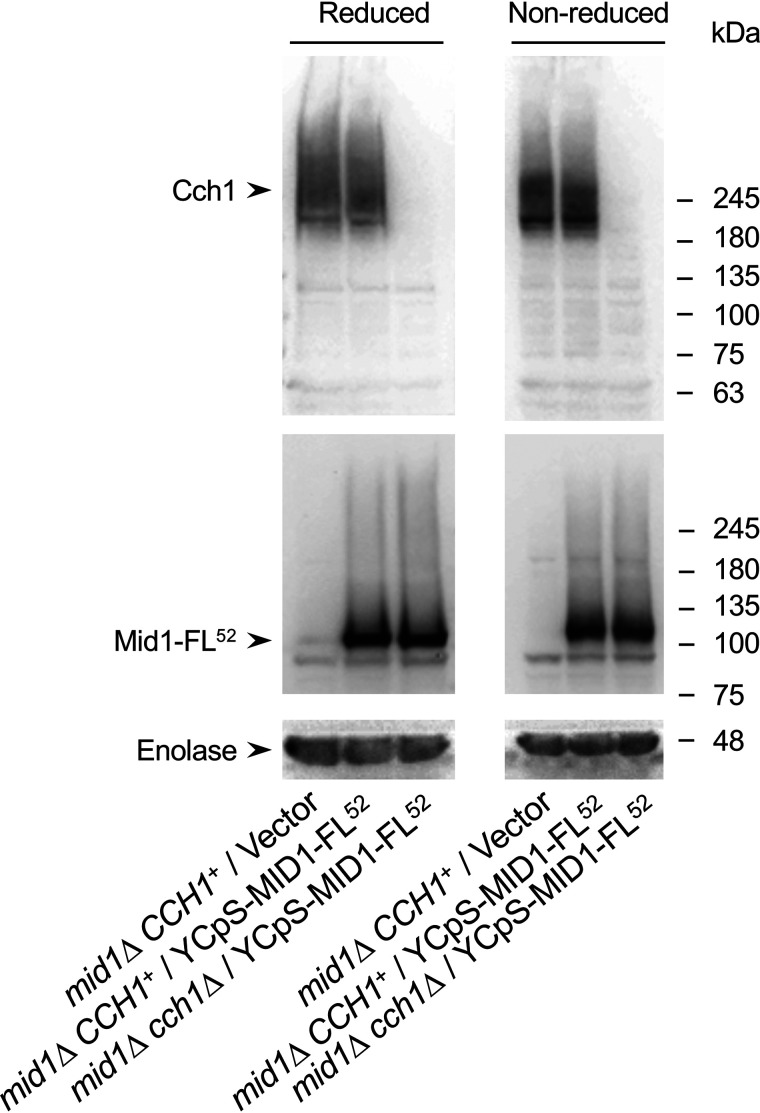
**Cch1 and Mid1 do not interact through disulfide bonds.** Whole-cell extracts prepared from cells of the *mid1*Δ mutant transformed with the plasmid YCpS-MID1-FL^52^, and the *mid1*Δ *cch1*Δ mutant transformed with the plasmid YCpS-MID1-FL^52^, were treated with (reduced) or without (nonreduced) the reducing reagent β-mercaptoethanol prior to SDS-PAGE followed by Western blotting. Reduced and nonreduced samples are shown on the *left* and *right panels*, respectively. Note that the electrophoretic mobilities of Cch1 and Mid1-FL^52^ in the presence of both proteins (*middle lane* on each panel) were essentially the same as those in the absence of Mid1-FL^52^ (*left lane* on each panel) and Cch1 (*right lane* on each panel). Also note that although a molecular mass marker with 300 kDa or greater is not employed, it is possible to guess that there is no Cch1/Mid1–FL^52^ complex with those sizes. Typical results from at least three independent experiments are shown.

Because Cys residues of both Cch1 and Mid1 were shown to be involved in the Cch1–Mid1 interaction, we can speculate that Cys residues may form intermolecular disulfide bonds that anchor the two subunits. To examine this possibility, we performed nonreducing SDS-PAGE followed by Western blotting. Whole-cell extracts prepared from the various yeast strains described in the legend to Fig. 8 were subjected to SDS-PAGE with or without the reducing reagent β-mercaptoethanol, and then to Western blotting using antibodies detecting Cch1 and FLAG-tagged Mid1. If the hypothesis is correct, a band corresponding to ∼300 kDa should be seen on the Western blotting because the monomeric forms of Cch1 and Mid1-FL^52^ are ∼200 and ∼100 kDa, respectively. However, a band corresponding to ∼300 kDa or greater was not detected on the blot (Fig. 8). This result is not conclusive, but consistent with the idea that Cys-1369 and/or Cys-1379 of Cch1 and Cys-498 of Mid1 are not engaged in intermolecular disulfide bonding.

## Discussion

### Cys-1369, Asp-1371, and Cys-1379 within Cch1 L5_III_ are important for its interaction with Mid1

In the present study, we identified extracellular amino acid residues involved in anchoring the interaction between the pore-forming subunit Cch1 and the regulatory subunit Mid1 in *S. cerevisiae*. This identification is important because both subunits are indispensable for constituting the functional Ca^2+^ channel required for the response to a variety of environmental stresses in yeasts. The alanine scanning mutagenesis experiments showed that among the eight highly conserved extracellular Cys residues of Cch1, only C1369A and C1379A mutations in L5_III_ abolished the interaction with Mid1 (Fig. 3, *C* and *D*). Other mutations (C587A, C606A, C636A, and C642A) of Cys residues present in L5_I_, and C1727A and C1738A present in L6_IV_, did not affect the physical interaction with Mid1, although these six mutations resulted in the functional loss of Ca^2+^ influx activity. In addition, the random mutagenesis experiments revealed that Asp-1371 present between Cys-1369 and Cys-1379 in L5_III_ is also required for the interaction with Mid1 (Fig. 4, *A* and *B*). These results suggest not only that the L5_III_ loop containing Cys-1369, Asp-1371, and Cys-1379 is involved in the interaction with Mid1, but also that the L5_I_ and L6_IV_ loops are unlikely to be involved in the interaction with Mid1, either alone or in pairs, because C1369A, D1371V, and C1379A mutants lacking Mid1-interacting activity possess intact L5_I_ and L6_IV_ loops. We, therefore, predict that the contributions of the L5_I_ and L6_IV_ loops to the interaction with Mid1 are minimal, if any.

### L5_III_ of Cch1/VGCC family members is important for physical interaction with Mid1 and the α_2_/δ subunit

Our finding that the L5_III_ loop of Cch1 is involved in its physical interaction with Mid1 is consistent with the results of a recent single-particle cryo-EM study, in which it was revealed that together with L5_II_ and L1-2_I_, L5_III_ of rabbit Ca_v_1.1 (α_1_ subunit) constitutes a docking site for the von Willebrand factor A domain of the α_2_/δ subunit (23, 24). Thus, the importance of L5_III_ in subunit interactions could be ubiquitous in all Cch1/Mid1 channels and VGCCs. Amino acid sequence alignment of L5_III_ supports this speculation; the primary sequence is significantly conserved from yeast Cch1 to mammalian VGCCs and NALCNs (Fig. 4*E*). In particular, Cys-1369, Asp-1371, and Cys-1379, identified as functionally essential residues in the present study, are completely conserved. Therefore, it is possible that L5_III_ plays a universal role in the physical interaction with Mid1 and/or the α_2_/δ subunit. However, this suggestion cannot be simply applied to the interaction between animal α_1_ subunits and their regulatory subunits. It is reported that the L1-2_I_ loop of the Ca_v_1.2 α_1_ subunit interacts with Ca_v_1.2 α_2_/δ subunit more substantially than L5_III_ (34), and that only the IIS5-S6 region including L5_II_ and L6_II_ of a NALCN homolog interacts with its regulatory subunit NLF-1 in *Caenorhabditis elegans* (19). Therefore, we speculate that the functional importance of L5_III_ differs between members of the Cch1/VGCC/NALCN family, even though the three key amino acid residues in L5_III_ are conserved. The difference may be attributed to structural differences between Mid1, the α_2_/δ subunit, and NLF1.

### Cys-498 of Mid1 is important for interaction with Cch1

Our present study showed that although 12 Cys residues in the Cys-rich domain of Mid1 are highly conserved in fungal Mid1 proteins, only three (Cys-417, Cys-431, and Cys-498) were required for *mid1*Δ-complementation activity, and only one (Cys-498) was involved in the interaction with Cch1. Our previous study showed that Cys-417 and Cys-431 are not included in the Frizzled-like motif (16) (Fig. 5*A*), and are specifically present in fungal Mid1 proteins and animal Mid1-related proteins, such as the NALCN-associated proteins dmeMid1 (CG33988) in *Drosophila melanogaster* and NFL-1 in *C. elegans*, and vertebrate FAM115A and B proteins homologous to NFL-1 (17, 35) (Fig. 6*D*). By contrast, Cys-498 is conserved not only in these proteins, but also in the extracellular Cys-rich domains of Frizzled proteins (17, 35).

Although the *S. cerevisiae* Mid1 protein has some structural features that are similar to those of animal α_2_/δ subunits (15, 16), the amino acid sequences are not homologous. In particular, our BLAST searches (https://blast.ncbi.nlm.nih.gov/Blast.cgi) and PHD secondary structure prediction (36) did not identify any regions that are homologous to the α_2_/δ von Willebrand factor A or Cache domains, both of which are binding sites for the α_1_ subunit (23, 24). Moreover, Mid1 (548 amino acids) is approximately half the size of the α_2_/δ subunit, and mutant Mid1 proteins N terminally truncated up to 209 amino acid residues still possess the ability to activate Cch1 (16). These features make it difficult to define a potential Cch1-interacting region in Mid1 based on information for the rabbit Ca_v_1.1 α_2_/δ subunit. Thus, we focused on the C-terminal Cys-rich domain because it is believed to be functionally important, and because this domain is highly conserved not only in fungal Mid1 homologs, but also in animal Mid1-related proteins, as described above. Furthermore, this domain resembles those of the Cys-rich domains of Frizzled receptors and Hedgehog-interacting proteins (35). However, it should be noted that the positioning pattern of Cys residues in this domain is not similar to that of Cys residues in the VGCC α_2_/δ subunit (17, 35), suggesting that the role of these Cys residues differs between Mid1 family proteins and the α_2_/δ subunit, in which they form disulfide bonds connecting the α_2_ subunit and the δ subunit, and engage in intramolecular disulfide bonds (23, 24).

The Cys-498 residue is located 51 amino acid residues from the C terminus of Mid1, in a random coil following an α-helix, as predicted by both PHD (36) and YASPIN (37) secondary structure prediction programs. The residue immediately following Cys-498 is Pro-499, which is highly conserved in fungal Mid1 and animal Mid1-related proteins. Because proline contributes to introduce a sharp turn in the protein structure, and is usually located on the protein surface (38), Cys-498 could be present near a turn on the surface of Mid1, where it may enable Mid1 to interact with Cch1.

### Cch1 and Mid1 do not appear to interact via disulfide bonding

Because Cch1 residues Cys-1369 and Cys-1379 and Mid1 residue Cys-498 are involved in interactions between the two subunits, Cch1 and Mid1 could potentially be connected by a disulfide bond. However, this is unlikely for two reasons; our SDS-PAGE analysis under nonreducing conditions did not reveal a band with a size equivalent to the Cch1–Mid1 complex (Fig. 8), and 3D cryo-EM and chemical cross-linking observations have shown that extracellular Cys residues of the rabbit Ca_v_1.1 α_1_ subunit participate in forming intraloop disulfide bonds that stabilize their respective loops (23, 24). Our transmembrane topology model and the positions of the eight conserved extracellular Cys residues of Cch1 are consistent with those of the rabbit Ca_v_1.1 α_1_ subunit. This implies that Cys-587–Cys-636, Cys-606–Cys-642, Cys-1369–Cys-1379, and Cys-1727–Cys-1738 intraloop disulfide bonds could be formed in Cch1, which are equivalent to those in rabbit Ca_v_1.1 (Cys-226–Cys-254, Cys-245–Cys-261, Cys-957–Cys-968, and Cys-1338–Cys-1352, respectively) (24). Therefore, it is unlikely that the Cys residues of Cch1 take part in intermolecular disulfide bonding. The possible contribution of Cys-1369 and Cys-1379 to intraloop disulfide bonding suggests that the C1369A and C1379A substitutions disrupt the L5_III_ loop structure, leading to inability of the loop to interact with Mid1.

In summary, we identified extracellular amino acid residues including Cys-1369, Asp-1371, and Cys-1379 located in L5_III_ of Cch1 that are involved in the interaction with Mid1, and amino acid residue Cys-498 of Mid1 that engages in the interaction with Cch1. The results suggest that the yeast Ca^2+^ channel is associated with both conserved and unique subunit interaction mechanisms. Specifically, the involvement of L5_III_ in the interaction with the regulatory subunit Mid1 and/or the α_2_/δ subunit appears to be conserved during molecular evolution of this channel family, whereas the region in Mid1 required for interaction with Cch1 appears to be unique. This uniqueness could be attributed to the structural divergence of Mid1 and the α_2_/δ subunit. The findings of the present study serve as a framework for further investigation of the *S. cerevisiae* Ca^2+^ channel, and for fungal and animal Ca^2+^ channels related to the Cch1/Mid1 channel.

## Experimental procedures

### Yeast strains

The parental strain H207 and its isogenic derivatives are described in Table 1.

**Table 1:** **Yeast strains used in this study**

Strain name	Genotype	Source
H207	*MAT***a** *his3-*Δ*1 leu2-3, 112 trp1-289 ura3-52 sst1-2*	7
H311	*mid1-*Δ*5*::*HIS3* in H207	39
H317	*cch1*Δ*::HIS3* in H207	28
H319	*mid1*Δ::*HIS3 cch1*Δ::*HIS3* in H207	40
MiF317	*MID1-5xFLAG*^52^ in H317	This study

### Media for yeast

Synthetic defined minimal medium containing 100 μm CaCl_2_ (SD.Ca100) and synthetic complete medium (SC) were prepared as described previously (7, 16). All yeast strains were cultured in SD.Ca100 medium for measurement of Ca^2+^ accumulation and cell viability or in SC medium for the preparation of cells extracts for Western blotting and co-immunoprecipitation experiments.

### Plasmids

Plasmids used in this study are listed in Table 2. Standard methods were used for manipulation of genetic materials (41). PCR-based site-directed mutagenesis was performed with a KOD plus mutagenesis kit (Toyobo Co., Ltd, Osaka, Japan) according to the manufacturer's protocol.

**Table 2:** **Plasmids used in this study**

Plasmid name	Genotype	Source
YCplac111	*LEU2 amp*^r^ ColE1-*ori ARS1 CEN4*	28
YCplac33	*URA3 amp*^r^ ColE1-*ori ARS1 CEN4*	40
YCpT-MID1_Xho_	*TDH3*p:*MID1*:*MID1*t in YCplac111	This study
YCpT-MID1_Xho_(C417A)*^a^*	*MID1*(C417A) in YCpT-MID1_Xho_	This study
YCpS-MID1-FL^52^[*LEU2*]	*MID1*p:*MID1-5xFLAG*^52^:*ADH1t* in YCplac111	16
YCpS-MID1-FL^52^[*URA3*]	*MID1*p:*MID1-5xFLAG*^52^:*ADH1t* in YCplac33	This study
pBC111	*LEU2 amp^r^ ColE1-ori-rop ARS1 CEN4*	28
pBCS-CCH1H	*CCH1*p:*CCH1*:*CCH1*t in pBC111	28
pBCT-CCH1H	*TDH3*p:*CCH1*:*ADH1*t in pBC111	28
pBCT-CCH1H-EGFP	*TDH3*p:*CCH1*:*EGFP*:*ADH1*t in pBC111	28
YCp-CCH1H_KpnSal_*^b^*	*CCH1H*_KpnSal_ (part of *CCH1* ORF; from 4,016 to the stop codon) in YCplac111	This study
pBCS-CCH1H_IIISacII_*^b^*	*CCH1*p:*CCH1H*_IIISacII_ (*Sac*II at 4,837):*ADH1*t in pBC111	This study

*^a^*A typical example of the plasmids carrying mutations in the *MID1* or *CCH1* gene. For detailed information about the other mutations, see the respective figures.

*^b^*500 bp upstream from the AUG codon was defined as position 1.

### Measurement of Ca^2+^ accumulation and viability of cells exposed to α-factor

Previously described methods were generally followed for Ca^2+^ accumulation and cell viability experiments (7, 42). For Ca^2+^ accumulation assays, cells were grown exponentially to a density of ∼2 × 10^6^ cells/ml in SD.Ca100 medium (1 ml) at 30˚C and received 6 μm α-factor with 185 kBq/ml (1.85 kBq/nmol) of ^45^CaCl_2_ (PerkinElmer Japan, NEZ013, Yokohama, Japan). Immediately after the addition of these compounds, an aliquot (100 µl; duplicate) was taken and filtered on a Millipore filter (type HA, pore size: 0.45 μm, Merck, Tokyo, Japan) presoaked in 5 mm CaCl_2_, and washed 5 times with 5 ml of the same solution. The remaining culture (0.8 ml) was incubated for 2 h and an aliquot (100 µl; duplicate) was processed as described above. The radioactivity retained on the filters was counted with a scintillation mixture, Emulsifier-Safe (PerkinElmer) in a liquid scintillation counter (Beckman Coulter LS6500, Tokyo, Japan).

For cell viability assays, cells were grown as described above, received 6 μm α-factor, and were incubated for 8 h. At 0 and 8 h after exposure to α-factor, the cells were examined for viability by the methylene blue method (42). Note that viable and dead cells are methylene blue-negative and -positive, respectively. The percentage of viability was calculated by dividing the number of methylene blue-negative cells by the total number of methylene blue-negative and -positive cells.

### Error-prone Mn-PCR

To introduce random mutations in the IIIS3−S6 region of Cch1, an error-prone random mutagenesis method with MnCl_2_ was employed (32). The IIIS3−S6 region of the plasmid YCp-CCH1H_KpnSal_ (100 ng, 2.0 ng/µl) was amplified in a reaction mixture containing 1.0 μm each of primer set CCH1-4314F (5′-GCTTACCTGAGGAATCCATGGAAC-3′) and CCH1-4965R (5'-CCTCAATGGTAAAGTAAGCGCTTC-3′), Ex Taq DNA polymerase (RR001A, Takara Bio Inc., Shiga, Japan), Ex Taq buffer (Takara Bio Inc.), 2.5 mm MgCl_2_, 0.4 mm dNTPs, and 100 μm MnCl_2_. To incorporate the resulting products in the *cch1* gene lacking a part of the IIIS3−S6 region, we employed the *in vivo* homologous recombination method (30), which enabled us to obtain plasmids carrying the entire sequence of the *cch1* genes. Using this method, error-prone Mn-PCR products were co-introduced into the *cch1*Δ mutant (∼4 × 10^7^ cells) with plasmid pBCS-CCH1H_IIISacII_, which had been predigested with ApaI, which cleaves an endogenous restriction site at position 4441 (500 bp upstream from the AUG codon was defined as position 1), and SacII (introduced by KOD plus mutagenesis at position 4837). Note that the two ends of the PCR products were homologous to the corresponding ends of the ApaI- and SacII-digested plasmid. From this co-transformation experiment we obtained ∼8,000 transformants, some of which were expected to contain the recombined plasmids carrying a mutation in the IIIS3−S6 region. To identify these plasmids, we performed five consecutive screening steps (see the next section).

### Selection of plasmids carrying cch1 genes mutated by error-prone Mn-PCR

In the first screening step, we directly examined the cell viability of the abovementioned transformants on transformation plates overlaid with soft agar containing α-factor using the methylene blue plate method (7), which enabled us to distinguish methylene blue-positive colonies composed mostly of dead cells from methylene blue-negative colonies composed mostly of viable cells. Using the methylene blue plate method, we selected 700 transformants.

In the second screening step, we inoculated the 700 transformants in 0.1 ml of SD.Ca100 medium (without α-factor) in 96-well–plates and incubated them overnight to reach stationary phase, during which cells with the *cch1* mutation lost viability and became methylene blue-positive. A total of 54 transformants with ≤30% viability were selected for further screening.

In the third screening step, we inoculated the 54 transformants in 2.0 ml of SD.Ca100 medium, incubated them for 6 h to log phase, and further incubated them for an additional 8 h in the presence of α-factor. A total of 51 transformants with ≤30% viability were selected for the fourth screening step.

In the fourth screening step, to eliminate transformants bearing *cch1* genes with either frameshift mutations or stop codons, as well as transformants expressing the Cch1 protein at low levels, we examined the 51 transformants for expression of the Cch1 protein by Western blotting with rabbit polyclonal anti-Cch1 antibodies (28). This screening step enabled us to select 31 transformants, each of which was expected to carry a plasmid harboring the *cch1* gene to produce a full-length mutant Cch1 protein at WT expression levels.

In the final screening step, plasmids were isolated from the 31 transformants and re-introduced into host strain H317. The resulting transformants were examined for viability after 8-h exposure to α-factor and eight transformants were selected. The plasmids from the eight transformants were subjected to DNA sequencing to confirm the mutated positions in Cch1.

### SDS-PAGE and Western blotting

SDS-PAGE and Western blotting were performed according to the methods described previously (7). To detect Cch1 and its derivatives or Mid1-FL^52^ and its derivatives on Western blots, rabbit polyclonal anti-Cch1 antibody (28) or mouse anti-DYKDDDDK mAb recognizing the FLAG tag (012-22384, Fujifilm Wako Pure Chemical Corp., Osaka, Japan) was employed as the first antibody. Enolase was employed as an internal control of protein abundance, and detected by rabbit polyclonal anti-enolase antibody (43). These antibodies were detected by horseradish peroxidase-conjugated donkey anti-rabbit IgG (NA934, GE Healthcare Japan, Tokyo, Japan) or horseradish peroxidase-conjugated sheep anti-mouse IgG (NA931, GE Healthcare Japan). Protein molecular weight markers (BlueStar Plus, Nippon Genetics, Tokyo, Japan; Rainbow, GE Healthcare Japan, Tokyo, Japan; or Kaleidpscope, Bio-Rad, Hercules, CA) were run on SDS-PAGE alongside the samples.

### Co-immunoprecipitation

For co-immunoprecipitation, a previously described method was followed, but membrane preparation was omitted (44). Cells expressing both Cch1 derivatives and Mid1-FL^52^ derivatives (∼3 × 10^7^ cells) grown in SC medium were harvested, washed once with Milli-Q water, resuspended in IP buffer (50 mm Tris-HCl, pH 8.0, 1.0% Triton X-100, 150 mm NaCl, 2 mm EDTA, 1 mm phenylmethylsulfonyl fluoride) containing 1 tablet/10 ml of cOmplete protease inhibitor mixture tablet (04693159001, Roche, Basel, Switzerland), and lysed with glass beads by vortexing. The lysate was combined with a mixture of either anti-Cch1 antibody (28) or anti-FLAG antibody (Fujifilm Wako Pure Chemical Corp.) and nProtein A-Sepharose 4 Fast Flow resin (17520001, GE Healthcare), incubated for 1 h at 4 °C with rotation, and centrifuged for 1 min at 12,000 × g. The pellet was washed three times with IP buffer by centrifugation. The final pellet was dissolved in SDS sample buffer (50 mm Tris-HCl, pH 6.8, 4% SDS, 10% glycerol, 0.05% bromphenol blue, 5% β-mercaptoethanol), heated for 5 min at 75 °C, and centrifuged for 5 min at 4 °C and 20,000 × *g*. The supernatant was subjected to SDS-PAGE followed by Western blotting.

### Confocal fluorescence microscopy

The method for confocal fluorescence microscopy was described previously (39). Briefly, cells expressing Cch1-EGFP or its derivatives were grown to a density of 6 × 10^6^ cells/ml, sonicated briefly to dissociate cell clumps, and placed on poly-l-lysine–coated glass coverslips. EGFP images were obtained at an excitation wavelength of 488 nm using an LSM780 confocal fluorescence microscope (Carl Zeiss MicroImaging, Tokyo, Japan).

### Indirect immunofluorescence microscopy

Indirect immunofluorescence microscopy was employed as described previously (45), except that mouse anti-DYKDDDDK antibody (Fujifilm Wako Pure Chemical Corp.) and Alexa Fluor 488-conjugated goat anti-mouse IgG (Invitrogen, A11029) were used as the first and second antibodies, respectively. Briefly, cells expressing Mid1-FL^52^ or its derivatives were grown to a density of 2 × 10^6^ cells/ml, sonicated briefly to dissociate cell clumps, concentrated, and placed on poly-l-lysine–coated glass coverslips. Cells were permeabilized and treated with the first antibody, followed by the second antibody. Alexa Fluor 488 images were obtained at an excitation wavelength of 488 nm using a TPC-SPE confocal fluorescence microscope (Leica Microsystems Japan, Tokyo).

### Statistical analysis

Statistical significance of differences was evaluated using ordinary one-way or two-way ANOVA followed by Dunette's post hoc test. Statistical analysis was performed with the multcomp package (version 1.4-13: R version 3.6.2) (46). Significance in difference between each single sample mean and a reference value was tested by the one sample *t test* using the stats package (part of R version 3.6.2) (47). Significant differences were accepted at *p* < 0.05.

## Data availability

All data are available in the main text or the supporting information.

## Supplementary Material

Supporting Information
