# Supplementary material for: Highly conserved extracellular residues mediate interactions between pore-forming and regulatory subunits of the yeast Ca2+ channel related to the animal VGCC/NALCN family
Source: J Biol Chem. 2020 Jul 20;295(37):13008–22. doi: 10.1074/jbc.RA120.014378 (PMC7489899; doi:10.1074/jbc.RA120.014378)
Supplement: Supporting Information [file supp_295_37_13008__index.html]

Highly conserved extracellular residues mediate interactions between pore-forming and regulatory subunits of the yeast Ca2+ channel related to the animal VGCC/NALCN family — Extracellular residues required for Cch1–Mid1 interaction — Supporting Information 

# Highly conserved extracellular residues mediate interactions between pore-forming and regulatory subunits of the yeast Ca2+ channel related to the animal VGCC/NALCN family

## Supporting Information

- Supporting Information (to be published online) - Supporting Information
