# Supplementary material for: Highly conserved extracellular residues mediate interactions between pore-forming and regulatory subunits of the yeast Ca2+ channel related to the animal VGCC/NALCN family
Source: J Biol Chem. 2020 Jul 20;295(37):13008–22. doi: 10.1074/jbc.RA120.014378 (PMC7489899; doi:10.1074/jbc.RA120.014378)
Supplement: Supporting Information [file supp_RA120.014378_160838_2_supp_564553_qdgpn4.pptx]

## Slide 1
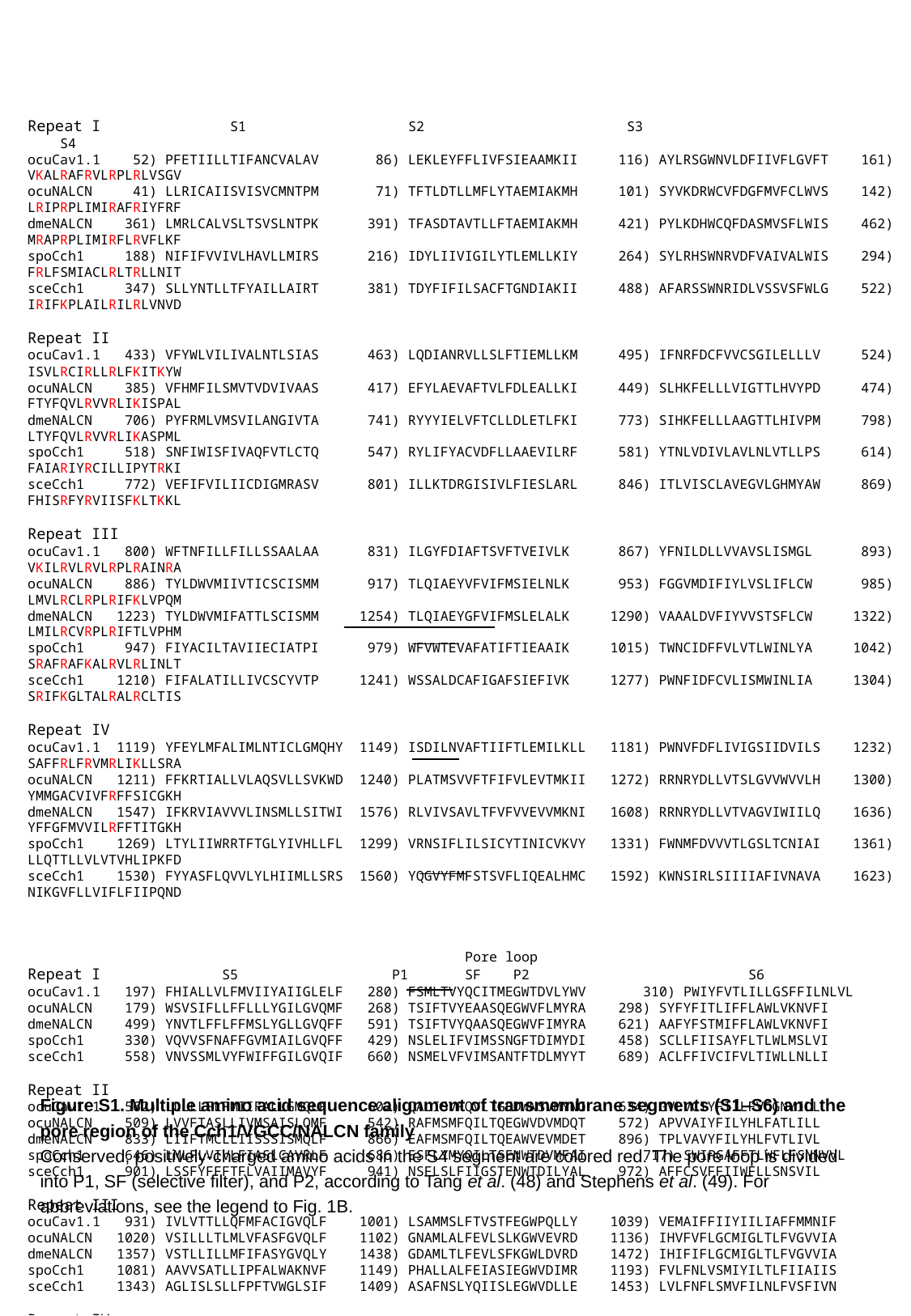

Repeat I S1		 S2	 S3		 S4
ocuCav1.1 52) PFETIILLTIFANCVALAV 86) LEKLEYFFLIVFSIEAAMKII 116) AYLRSGWNVLDFIIVFLGVFT 161) VKALRAFRVLRPLRLVSGV
ocuNALCN 41) LLRICAIISVISVCMNTPM 71) TFTLDTLLMFLYTAEMIAKMH 101) SYVKDRWCVFDGFMVFCLWVS 142) LRIPRPLIMIRAFRIYFRF
dmeNALCN 361) LMRLCALVSLTSVSLNTPK 391) TFASDTAVTLLFTAEMIAKMH 421) PYLKDHWCQFDASMVSFLWIS 462) MRAPRPLIMIRFLRVFLKF
spoCch1 188) NIFIFVVIVLHAVLLMIRS 216) IDYLIIVIGILYTLEMLLKIY 264) SYLRHSWNRVDFVAIVALWIS 294) FRLFSMIACLRLTRLLNIT
sceCch1 347) SLLYNTLLTFYAILLAIRT 381) TDYFIFILSACFTGNDIAKII 488) AFARSSWNRIDLVSSVSFWLG 522) IRIFKPLAILRILRLVNVD
Repeat II
ocuCav1.1 433) VFYWLVILIVALNTLSIAS 463) LQDIANRVLLSLFTIEMLLKM 495) IFNRFDCFVVCSGILELLLV 524) ISVLRCIRLLRLFKITKYW
ocuNALCN 385) VFHMFILSMVTVDVIVAAS 417) EFYLAEVAFTVLFDLEALLKI 449) SLHKFELLLVIGTTLHVYPD 474) FTYFQVLRVVRLIKISPAL
dmeNALCN 706) PYFRMLVMSVILANGIVTA 741) RYYYIELVFTCLLDLETLFKI 773) SIHKFELLLAAGTTLHIVPM 798) LTYFQVLRVVRLIKASPML
spoCch1 518) SNFIWISFIVAQFVTLCTQ 547) RYLIFYACVDFLLAAEVILRF 581) YTNLVDIVLAVLNLVTLLPS 614) FAIARIYRCILLIPYTRKI
sceCch1 772) VEFIFVILIICDIGMRASV 801) ILLKTDRGISIVLFIESLARL 846) ITLVISCLAVEGVLGHMYAW 869) FHISRFYRVIISFKLTKKL
Repeat III
ocuCav1.1 800) WFTNFILLFILLSSAALAA 831) ILGYFDIAFTSVFTVEIVLK 867) YFNILDLLVVAVSLISMGL 893) VKILRVLRVLRPLRAINRA
ocuNALCN 886) TYLDWVMIIVTICSCISMM 917) TLQIAEYVFVIFMSIELNLK 953) FGGVMDIFIYLVSLIFLCW 985) LMVLRCLRPLRIFKLVPQM
dmeNALCN 1223) TYLDWVMIFATTLSCISMM 1254) TLQIAEYGFVIFMSLELALK 1290) VAAALDVFIYVVSTSFLCW 1322) LMILRCVRPLRIFTLVPHM
spoCch1 947) FIYACILTAVIIECIATPI 979) WFVWTEVAFATIFTIEAAIK 1015) TWNCIDFFVLVTLWINLYA 1042) SRAFRAFKALRVLRLINLT
sceCch1 1210) FIFALATILLIVCSCYVTP 1241) WSSALDCAFIGAFSIEFIVK 1277) PWNFIDFCVLISMWINLIA 1304) SRIFKGLTALRALRCLTIS
Repeat IV
ocuCav1.1 1119) YFEYLMFALIMLNTICLGMQHY 1149) ISDILNVAFTIIFTLEMILKLL 1181) PWNVFDFLIVIGSIIDVILS 1232) SAFFRLFRVMRLIKLLSRA
ocuNALCN 1211) FFKRTIALLVLAQSVLLSVKWD 1240) PLATMSVVFTFIFVLEVTMKII 1272) RRNRYDLLVTSLGVVWVVLH 1300) YMMGACVIVFRFFSICGKH
dmeNALCN 1547) IFKRVIAVVVLINSMLLSITWI 1576) RLVIVSAVLTFVFVVEVVMKNI 1608) RRNRYDLLVTVAGVIWIILQ 1636) YFFGFMVVILRFFTITGKH
spoCch1 1269) LTYLIIWRRTFTGLYIVHLLFL 1299) VRNSIFLILSICYTINICVKVY 1331) FWNMFDVVVTLGSLTCNIAI 1361) LLQTTLLVLVTVHLIPKFD
sceCch1 1530) FYYASFLQVVLYLHIIMLLSRS 1560) YQGVYFMFSTSVFLIQEALHMC 1592) KWNSIRLSIIIIAFIVNAVA 1623) NIKGVFLLVIFLFIIPQND
 Pore loop
Repeat I	 S5	 P1 SF P2	 S6
ocuCav1.1 197) FHIALLVLFMVIIYAIIGLELF 280) FSMLTVYQCITMEGWTDVLYWV	 310) PWIYFVTLILLGSFFILNLVL
ocuNALCN 179) WSVSIFLLFFLLLYGILGVQMF 268) TSIFTVYEAASQEGWVFLMYRA 298) SYFYFITLIFFLAWLVKNVFI
dmeNALCN 499) YNVTLFFLFFMSLYGLLGVQFF 591) TSIFTVYQAASQEGWVFIMYRA 621) AAFYFSTMIFFLAWLVKNVFI
spoCch1 330) VQVVSFNAFFGVMIAILGVQFF 429) NSLELIFVIMSSNGFTDIMYDI 458) SCLLFIISAYFLTLWLMSLVI
sceCch1 558) VNVSSMLVYFWIFFGILGVQIF 660) NSMELVFVIMSANTFTDLMYYT 689) ACLFFIVCIFVLTIWLLNLLI
Repeat II
ocuCav1.1 562) LLLLLFLFMIIFALLGMQLF 602) QALISVFQVLTGEDWNSVMYNG 634) GVLVCIYFIILFVCGNYILL
ocuNALCN 509) LVVFTASLLIVMSAISLQMF 542) RAFMSMFQILTQEGWVDVMDQT 572) APVVAIYFILYHLFATLILL
dmeNALCN 833) LIIFTMCLLIISSSISMQLF 866) EAFMSMFQILTQEAWVEVMDET 896) TPLVAVYFILYHLFVTLIVL
spoCch1 646) LMLFLVIVLFIASLCAVRLF 686) ESFLYMYQILTSENWTDVMFAI	 717) SWIPGAFFTLWFLFSNNVVL
sceCch1 901) LSSFYFFFTFLVAIIMAVYF 941) NSFLSLFIIGSTENWTDILYAL 972) AFFCSVFFIIWFLLSNSVIL
Repeat III
ocuCav1.1 931) IVLVTTLLQFMFACIGVQLF 1001) LSAMMSLFTVSTFEGWPQLLY 1039) VEMAIFFIIYIILIAFFMMNIF
ocuNALCN 1020) VSILLLTLMLVFASFGVQLF 1102) GNAMLALFEVLSLKGWVEVRD 1136) IHVFVFLGCMIGLTLFVGVVIA
dmeNALCN 1357) VSTLLILLMFIFASYGVQLY 1438) GDAMLTLFEVLSFKGWLDVRD 1472) IHIFIFLGCMIGLTLFVGVVIA
spoCch1 1081) AAVVSATLLIPFALWAKNVF 1149) PHALLALFEIASIEGWVDIMR 1193) FVLFNLVSMIYILTLFIIAIIS
sceCch1 1343) AGLISLSLLFPFTVWGLSIF 1409) ASAFNSLYQIISLEGWVDLLE 1453) LVLFNFLSMVFILNLFVSFIVN
Repeat IV
ocuCav1.1 1269) YVALLIVMLFFIYAVIGMQMF 1312) AVLLLFRCATGEAWQEILL 1357) AYYYFISFYMLCAFLIINLFVAVIM
ocuNALCN 1337) FIIVGMFLLLLCYAFAGVVLF 1378) AITVLFRIVTGEDWNKIMH 1423) ALMYFCSFYVIIAYIMLNLLVAIIV
dmeNALCN 1673) FIIFGMFLLVFFYALAGTILF 1714) GVAMLFRIVTGEDWNKIMH 1758) SLIYFCTFYVIITYIVLNLLVAIIM
spoCch1 1387) SLIATWIVLYITFAIAFNQIF 1438) ALVLLFTMTFGEGWNDVMH 1482) AYGLFIAWNIISMYIFVNMFITVVF
sceCch1 1659) SLTYTWGVLFLVYAIALNQIF 1700) SMIVLFRCSFGEGWNYIMA 1744) AYLLLMSWNIISMYIFVNMFVSLII
Figure S1. Multiple amino acid sequence alignment of transmembrane segments (S1–S6) and the pore region of the Cch1/VGCC/NALCN family
Conserved, positively-charged amino acids in the S4 segment are colored red. The pore loop is divided into P1, SF (selective filter), and P2, according to Tang et al. (48) and Stephens et al. (49). For abbreviations, see the legend to Fig. 1B.

## Slide 2
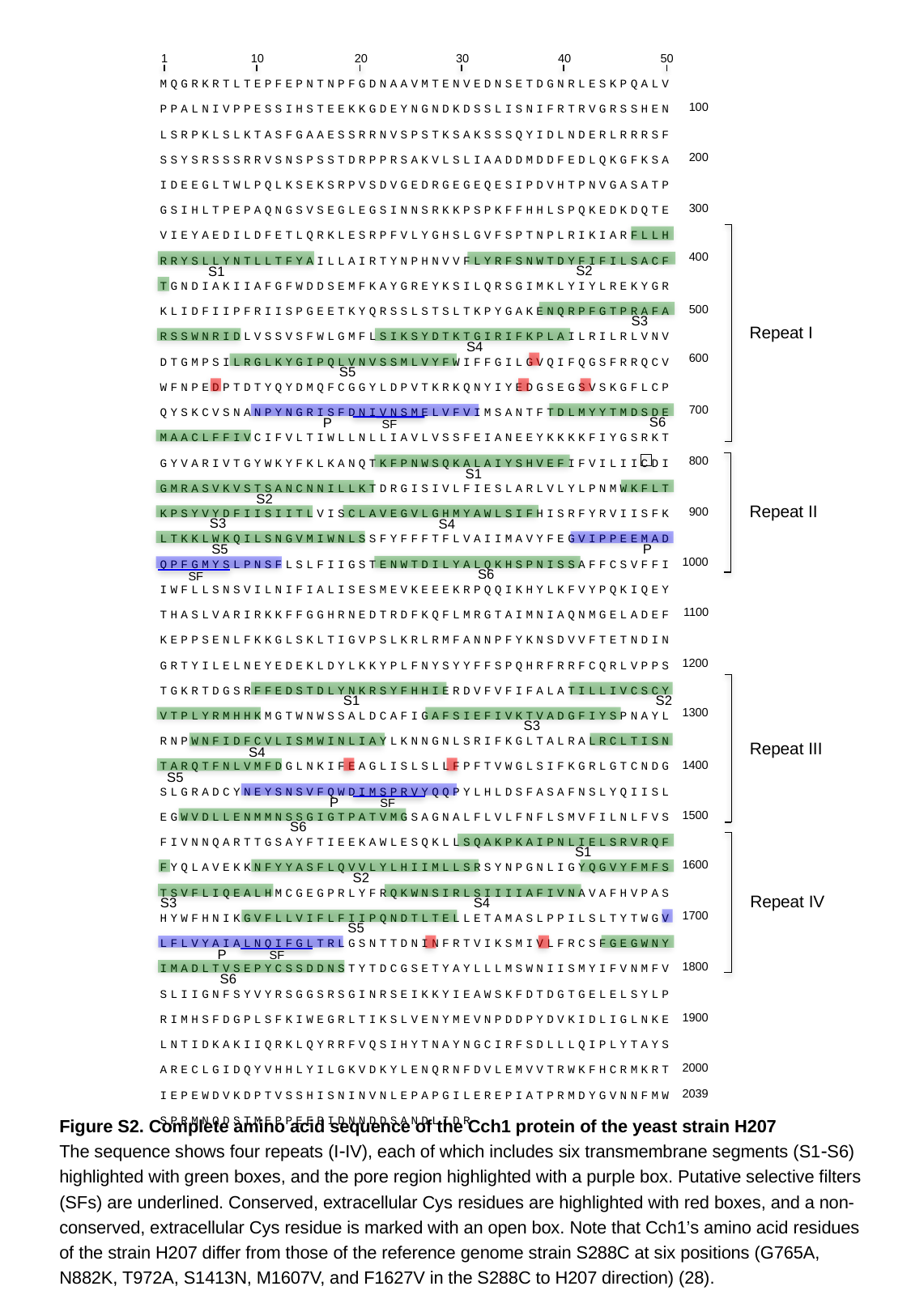

MQGRKRTLTEPFEPNTNPFGDNAAVMTENVEDNSETDGNRLESKPQALVPPALNIVPPESSIHSTEEKKGDEYNGNDKDSSLISNIFRTRVGRSSHENLSRPKLSLKTASFGAAESSRRNVSPSTKSAKSSSQYIDLNDERLRRRSFSSYSRSSSRRVSNSPSSTDRPPRSAKVLSLIAADDMDDFEDLQKGFKSAIDEEGLTWLPQLKSEKSRPVSDVGEDRGEGEQESIPDVHTPNVGASATPGSIHLTPEPAQNGSVSEGLEGSINNSRKKPSPKFFHHLSPQKEDKDQTEVIEYAEDILDFETLQRKLESRPFVLYGHSLGVFSPTNPLRIKIARFLLHRRYSLLYNTLLTFYAILLAIRTYNPHNVVFLYRFSNWTDYFIFILSACFTGNDIAKIIAFGFWDDSEMFKAYGREYKSILQRSGIMKLYIYLREKYGRKLIDFIIPFRIISPGEETKYQRSSLSTSLTKPYGAKENQRPFGTPRAFARSSWNRIDLVSSVSFWLGMFLSIKSYDTKTGIRIFKPLAILRILRLVNVDTGMPSILRGLKYGIPQLVNVSSMLVYFWIFFGILGVQIFQGSFRRQCVWFNPEDPTDTYQYDMQFCGGYLDPVTKRKQNYIYEDGSEGSVSKGFLCPQYSKCVSNANPYNGRISFDNIVNSMELVFVIMSANTFTDLMYYTMDSDEMAACLFFIVCIFVLTIWLLNLLIAVLVSSFEIANEEYKKKKFIYGSRKTGYVARIVTGYWKYFKLKANQTKFPNWSQKALAIYSHVEFIFVILIICDIGMRASVKVSTSANCNNILLKTDRGISIVLFIESLARLVLYLPNMWKFLTKPSYVYDFIISIITLVISCLAVEGVLGHMYAWLSIFHISRFYRVIISFKLTKKLWKQILSNGVMIWNLSSFYFFFTFLVAIIMAVYFEGVIPPEEMADQPFGMYSLPNSFLSLFIIGSTENWTDILYALQKHSPNISSAFFCSVFFIIWFLLSNSVILNIFIALISESMEVKEEEKRPQQIKHYLKFVYPQKIQEYTHASLVARIRKKFFGGHRNEDTRDFKQFLMRGTAIMNIAQNMGELADEFKEPPSENLFKKGLSKLTIGVPSLKRLRMFANNPFYKNSDVVFTETNDINGRTYILELNEYEDEKLDYLKKYPLFNYSYYFFSPQHRFRRFCQRLVPPSTGKRTDGSRFFEDSTDLYNKRSYFHHIERDVFVFIFALATILLIVCSCYVTPLYRMHHKMGTWNWSSALDCAFIGAFSIEFIVKTVADGFIYSPNAYLRNPWNFIDFCVLISMWINLIAYLKNNGNLSRIFKGLTALRALRCLTISNTARQTFNLVMFDGLNKIFEAGLISLSLLFPFTVWGLSIFKGRLGTCNDGSLGRADCYNEYSNSVFQWDIMSPRVYQQPYLHLDSFASAFNSLYQIISLEGWVDLLENMMNSSGIGTPATVMGSAGNALFLVLFNFLSMVFILNLFVSFIVNNQARTTGSAYFTIEEKAWLESQKLLSQAKPKAIPNLIELSRVRQFFYQLAVEKKNFYYASFLQVVLYLHIIMLLSRSYNPGNLIGYQGVYFMFSTSVFLIQEALHMCGEGPRLYFRQKWNSIRLSIIIIAFIVNAVAFHVPASHYWFHNIKGVFLLVIFLFIIPQNDTLTELLETAMASLPPILSLTYTWGVLFLVYAIALNQIFGLTRLGSNTTDNINFRTVIKSMIVLFRCSFGEGWNYIMADLTVSEPYCSSDDNSTYTDCGSETYAYLLLMSWNIISMYIFVNMFVSLIIGNFSYVYRSGGSRSGINRSEIKKYIEAWSKFDTDGTGELELSYLPRIMHSFDGPLSFKIWEGRLTIKSLVENYMEVNPDDPYDVKIDLIGLNKELNTIDKAKIIQRKLQYRRFVQSIHYTNAYNGCIRFSDLLLQIPLYTAYSARECLGIDQYVHHLYILGKVDKYLENQRNFDVLEMVVTRWKFHCRMKRTIEPEWDVKDPTVSSHISNINVNLEPAPGILEREPIATPRMDYGVNNFMWSPRMNQDSTMEPPEEPIDNNDDSANDLIDR
1
10
20
30
40
50
100
200
300
400
S2
S1
500
S3
Repeat I
S4
600
S5
700
S6
P
SF
800
S1
S2
Repeat II
900
S3
S4
S5
P
1000
S6
SF
1100
1200
S1
S2
1300
S3
Repeat III
S4
1400
S5
P
SF
1500
S6
S1
1600
S2
Repeat IV
S4
S3
1700
S5
P
SF
1800
S6
1900
2000
2039
Figure S2. Complete amino acid sequence of the Cch1 protein of the yeast strain H207
The sequence shows four repeats (I−IV), each of which includes six transmembrane segments (S1−S6) highlighted with green boxes, and the pore region highlighted with a purple box. Putative selective filters (SFs) are underlined. Conserved, extracellular Cys residues are highlighted with red boxes, and a non-conserved, extracellular Cys residue is marked with an open box. Note that Cch1’s amino acid residues of the strain H207 differ from those of the reference genome strain S288C at six positions (G765A, N882K, T972A, S1413N, M1607V, and F1627V in the S288C to H207 direction) (28).
